# “Resistance leads to self-destruction”: how an (a)political strategy helped Karl von Frisch succeed during the Nazi era

**DOI:** 10.1007/s00359-024-01697-3

**Published:** 2024-03-28

**Authors:** Günther K. H. Zupanc, Susanne Wanninger

**Affiliations:** 1 Laboratory of Neurobiology, Department of Biology, Northeastern University, Boston, MA 02115 USA; 2 Universitätsarchiv, Abteilung Historische Sammlungen, Universitätsbibliothek, Ludwig-Maximilians-Universität München, 80539 Munich, Germany

**Keywords:** Karl von Frisch, White Rose, Sophie Scholl, Konrad Lorenz, Niko Tinbergen, Werner Jacobs

## Abstract

Karl von Frisch, one of the leading zoologists of the twentieth century and co-founder of the Journal of Comparative Physiology A, has been frequently portrayed as an opponent of the Nazi regime because he, as a ‘quarter-Jew,’ faced the threat of forced retirement from his position as a professor at the University of Munich during the Third Reich. However, doubts about an active opposition role have surfaced in recent years. A litmus test for assessing the validity of this notion is provided by our discovery that four of the six core members of the anti-Nazi resistance group ‘White Rose’—Sophie Scholl, Hans Scholl, Christoph Probst, and Alexander Schmorell—were his students. When they were arrested, sentenced to death, and executed, he seemed to ignore this historic event, both during and after World War II—in line with his belief that resistance leads to self-destruction, and research can flourish only by ignoring what happens around oneself. On the other hand, this seemingly apolitical attitude did not prevent him from making use of politics when it served his interests. Such actions included his (pseudo-)scientific justification of forced sterilization of people suffering from hereditary disorders during the Third Reich and his praise of the Nazi government’s efforts to “keep races pure.” As unsettling as these and some other political views and actions of Karl von Frisch are, they enabled him to carry out several critical pieces of his research agenda during the Third Reich, which three decades later earned him a Nobel Prize.

## Introduction

**Fig. 1:**
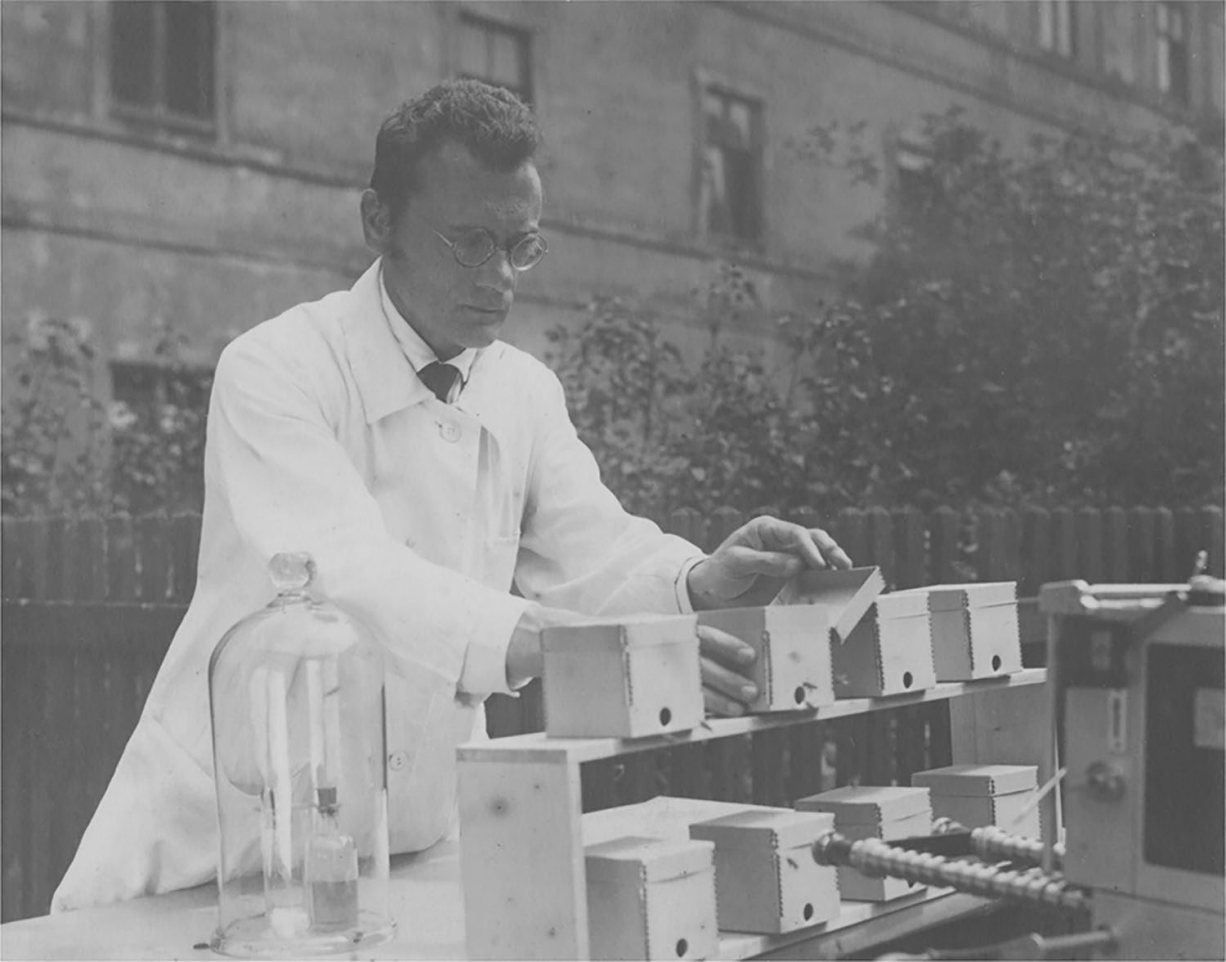
Karl von Frisch performing an odor experiment in the garden of the *Alte Akademie* (Old Academy) in Munich, around 1930. In this experiment, bees were trained to enter the inside of cardboard boxes through the round hole in front, where they learned to associate the presence of sugar water with a specific floral scent, such as jasmine oil. Courtesy: Bayerische Staatsbibliothek München/Bildarchiv

In 1924, Karl von Frisch (1886–1982), together with Alfred Kühn,^1^ founded the Journal of Comparative Physiology A, then under its German title *Zeitschrift für vergleichende Physiologie* (for a historical account see Zupanc 2022). He served as its Editor-in-Chief until 1960. For his pioneering research at the interface in ethology and sensory physiology, he shared the 1973 Nobel Prize in Physiology or Medicine with Konrad Lorenz^2^ and Niko Tinbergen.^3^ His major discoveries include hearing and *Schreckstoff*^4^ of fish, as well as color and polarization vision and the dance language in honeybees (Fig. 1).

Karl von Frisch’s name is inextricably linked to Munich and the University of Munich. From 1908 to 1909, he studied zoology there for three semesters under Richard Hertwig^5^; from 1910 to 1912, he was an assistant of Hertwig at the Zoological Institute of the University of Munich and, in 1925, he succeeded him as *Ordinarius*^6^; in 1946, he retired from his position but returned to the University of Munich in 1950; in 1958, he retired but continued research at the University as emeritus professor; in 1973, he received the Nobel Prize for Physiology or Medicine while he resided in Munich; and, in 1982, he died in Munich, where he is also buried.

While Karl von Frisch enjoyed his greatest successes in Munich, he also suffered the worst humiliation of his life during his tenure at the University of Munich. After two assistants of the Zoological Institute had denounced him as having a Jewish grandmother in 1936, he was categorized as a so-called ‘quarter-Jew.’^7^ In January 1941, he was informed by the *Bayerisches Staatsministerium für Unterricht und Kultus* (Bavarian State Ministry for Education and Culture) that, due to his ancestry, the Nazi *Reichsministerium für Wissenschaft, Erziehung und Volksbildung* (Reich Ministry of Science, Education, and National Culture) would seek his early retirement from his *Ordinarius* position.^8^ Karl von Frisch fought this decision and, with the help of some influential supporters (including Munich zoologists who signed a petition letter to the Reich Ministry of Science, Education, and National Culture), he finally succeeded, in 1942, in delaying forced retirement to lead the scientific efforts to combat the *Nosema* infection that raged across European bee colonies and resulted in devastating losses in the early 1940s (for review see Zupanc 2024 in this issue).

Given the threat by the Nazis that Karl von Frisch faced after 1936, it is surprising that he succeeded not only in keeping his position as *Ordinarius* and director of the Zoological Institute but also in making what perhaps were the most significant discoveries of his scientific career. These accomplishments are even more surprising, as he has been widely considered an “opponent of the Nazi regime” (Föger and Taschwer 2001, p. 158).^9^

A litmus test for assessing the validity of this notion was provided after we had discovered during the historical research presented here that four of the six core members of the anti-Nazi resistance group *Weiße Rose* (White Rose) were students of him; and made documented attempts to win over to their cause at least two of his associates, and perhaps von Frisch himself.

Surprisingly, however, we did not find any indication that he ever supported the White Rose, acknowledged their activism, or even just mentioned their existence. Yet, further research suggested that his supposed ignorance of White Rose as the most significant non-violent anti-Nazi resistance group aligns well with his lifelong determination to “stay out of politics” so that he could do research without any distraction. This attitude explains how, during the time when the war reached its most brutal phase and political oppression became worse than ever before, he succeeded in making what perhaps are the most impactful discoveries of his scientific career, ultimately establishing his fame as one of the leading zoologists of the twentieth century.

Paradoxically, his apolitical attitude did not prevent him from using politics, including the mobilization of the help of influential Nazis and the demonstration of loyalty to Hitler’s government, to advance his scientific agenda. His willingness to employ such a delicate strategy clearly shows that the picture of him as an opponent of the Nazi regime needs to be repainted.

## Karl von Frisch and the White Rose resistance group

### Historical background

Whereas the University of Munich was one of the leading German universities in science, particularly physics and chemistry, it was also a Nazi stronghold. As early as in 1925, Richard Willstätter^10^ resigned from his position as *Ordinarius* at the University as an expression of his protest over the refusal to appoint Victor Moritz Goldschmidt, a geochemist of Jewish heritage, to a professorship. Willstätter viewed this refusal as a sign of increasing anti-Semitism impacting faculty appointments.

While around that time the Nazi party was largely unknown outside of Munich, it was there that Adolf Hitler and the Nazis rose to power. Hitler had moved from Austria to Munich in 1913, and he officially remained a resident until his death. In 1923, at the *Bürgerbräukeller*, a beer hall in Munich, began what has become known as the Beer Hall Putsch—the (failed) attempt of Hitler and Erich Ludendorff,^11^ supported by several hundred members of the SA,^12^ to overthrow the German government. In recognition of the critical role that Munich had played in the rise of National Socialism, Hitler conferred in 1935, two years after he had become chancellor, on Munich the honorary title *Hauptstadt der Bewegung* (capital of the movement).

**Fig. 2:**
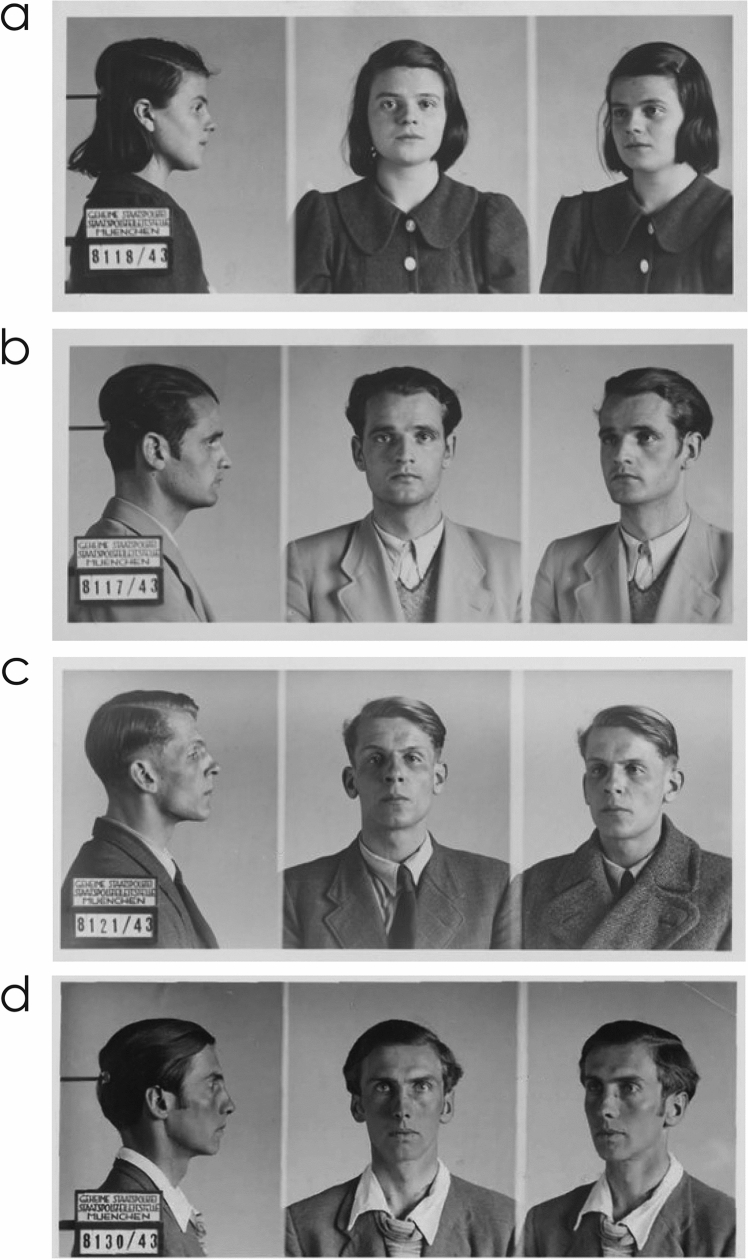
Members of the White Rose who took courses with Karl von Frisch at the University of Munich. **a** Sophie Scholl. **b** Hans Scholl. **c** Christoph Probst. **d** Alexander Schmorell. The photographs were taken after their arrests by the Gestapo in Munich. Courtesy: Stadtarchiv München **a**: reference number DE-1992-FS-PER-S-0008-01; **b**: reference number DE-1992-FS-PER-S-0007-01; **c**: reference number DE-1992-FS-PER-P-0003-01; Wikimedia Commons (**d**). Photographer(s) unknown

Despite its role during the rise of National Socialism and the time of the Nazi government, Munich was also the site of some powerful anti-Nazi resistance. The best-known is the non-violent activism of the White Rose resistance group that wrote and distributed leaflets in which they called for opposition to the Nazi regime and an end to the war. Its core consisted of five students—Sophie Scholl (1921–1943) (Fig. 2a), Hans Scholl (1918–1943) (Fig. 2b), Christoph Probst (1919–1943) (Fig. 2c), Alexander Schmorell (1917–1943) (Fig. 2d), and Willi Graf (1918–1943) —and one professor—Kurt Huber (1893–1943)—of the University of Munich. Their activities started in the summer of 1942 and ended beginning with their arrests, on February 18, 1943. Ultimately, all of them were executed. These six core members were supported by several dozens of other individuals, both in and outside of Munich.

Sophie Scholl^13^ majored in biology and minored in philosophy. All four male students went to medical school. Their study at the University alternated with periods of compulsory service in military hospitals at the front. Kurt Huber was a professor of philosophy and musicology. Motivated by humanistic ideals and, at least in part, religious beliefs, their writings were strongly influenced by the brutality of the war and the German atrocities (including the mass murder of Jews) that Willi Graf, Christoph Probst, Alexander Schmorell, and Hans Scholl witnessed on the Eastern Front.

The first four leaflets, entitled ‘Leaflets of the White Rose,’ were written by Alexander Schmorell and Hans Scholl in June and July 1942.^14^ The approximately 100 copies of each leaflet were sent primarily to the German intelligentsia, whom they called for resistance: “It is not only your right but also your moral obligation to remove the regime.”^15^

Towards the end of 1942, Sophie Scholl, Willi Graf, Christoph Probst, and Kurt Huber joined the group. Together they wrote the fifth leaflet in January 1943. It is entitled ‘Leaflets of the Resistance Movement in Germany.’ The sixth leaflet, titled ‘Fellow Students’ was mostly written by Kurt Huber and produced in February 1943. In the latter two leaflets, the authors changed the language to appeal to a larger segment of the population. They clearly spelled out their conviction that the war was lost (“Hitler cannot win the war; he can only prolong it”^16^) and urged the reader to “support the resistance movement.”^17^ Using better duplicating technology, they produced several thousand copies of each leaflet, which they distributed in Munich and sent by trusted couriers to other German cities.

**Fig. 3:**
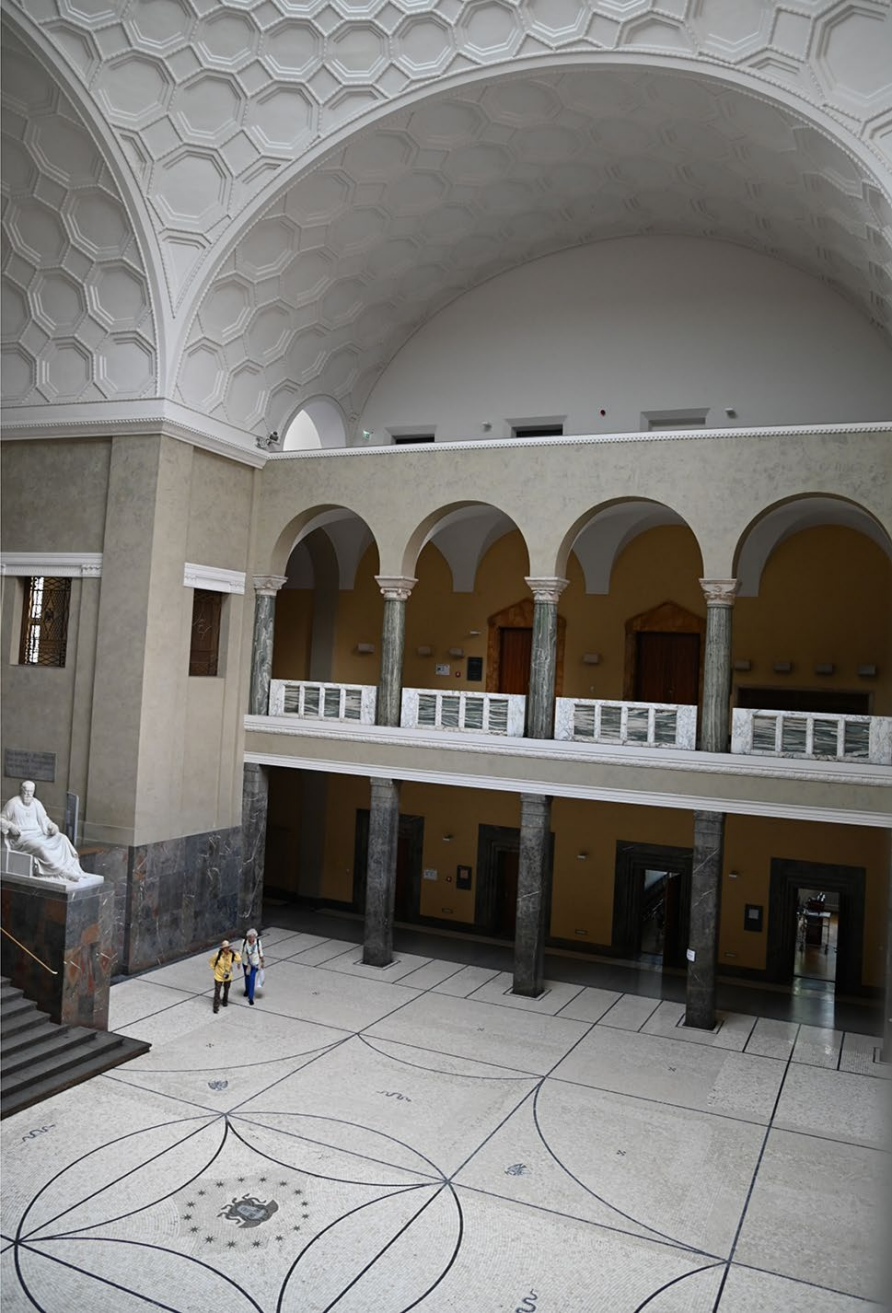
*Lichthof* (atrium) of the main building of the University of Munich. On February 18, 1943, after Hans and Sophie Scholl had distributed most of their copies of the sixth leaflet, Sophie flung the remaining copies from the top floor of the gallery down into the atrium. This was observed by a member of the maintenance staff, ultimately leading to the arrest of the Scholl siblings by the Gestapo. Photograph by Günther K.H. Zupanc

On the morning of February 18, 1943, Hans and Sophie Scholl took a suitcase full of copies of the sixth leaflet (and some copies of the fifth leaflet) to the main building of the University, where they left them in front of lecture halls so that students would find them after classes. Sophie Scholl then flung the remaining copies from the gallery of the top floor down into the atrium (Fig. 3). This was watched by a janitor, who took the Scholl siblings to the office of Ernst Haeffner, *Syndicus* (syndic) of the University, who called the Gestapo^18^ (Hockerts 2022).

Over the next days and months, all the remaining core members of the White Rose and many individuals associated with the group were apprehended. On February 22, 1943, the Scholl siblings and Christoph Probst stood trial before the First Senate of the *Volksgerichtshof* (‘People’s Court’) headed by Roland Freisler^19^ in Munich and were sentenced to death. They were executed the same day by guillotine in the Stadelheim Prison. In later trials, the other three core members of the group—Alexander Schmorell, Willi Graf, and Kurt Huber—were also sentenced to death and subsequently executed.

In total, 31 persons associated with the White Rose stood trial—the six core members plus 25 supporters.^20^ Many of the latter were given long-prison sentences. One of the supporters, Hans Conrad Leipelt (1921–1945), a ‘half-Jew’^21^ under the Nuremberg Laws on race of 1935 and a chemistry student at the University of Munich,^22^ was sentenced to death and executed. After the execution of the Scholl siblings and Christoph Probst, he had copied the sixth leaflet to which he added the title “… and yet their spirit lives on!” Supported by friends, he distributed this leaflet in Hamburg and Munich. When he collected money for the widow of Professor Kurt Huber, he was denounced, arrested, tried, and executed in 1945.

A report about the White Rose and the text of their sixth leaflet were smuggled out of Germany. This leaflet was retitled ‘The Manifesto of the Students of Munich,’ and millions of copies were dropped by Allied planes over Germany in July 1943.

### Enrollment of members of the White Rose in courses taught by Karl von Frisch

**Fig. 4:**
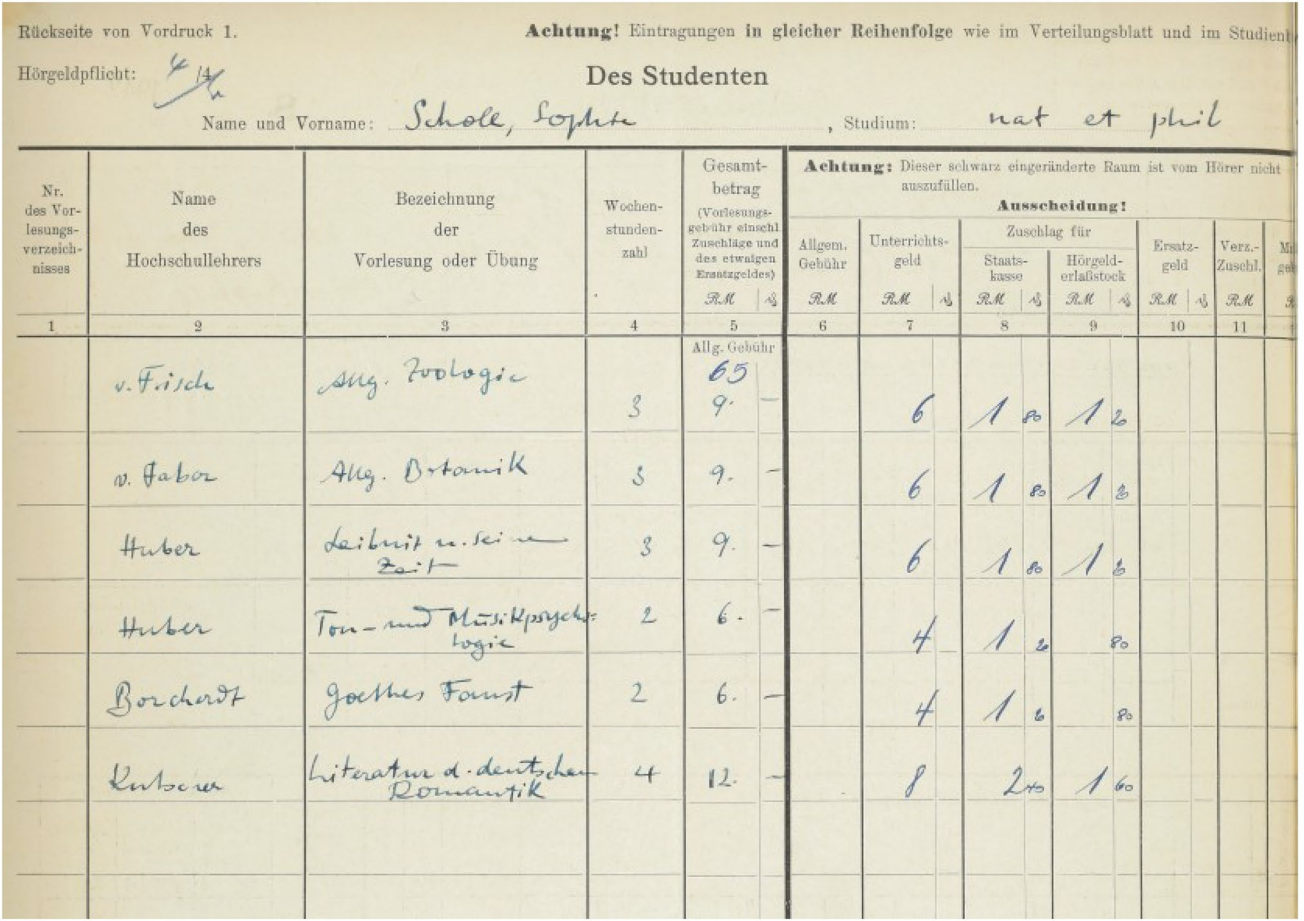
*Belegblatt* (course registration card) of Sophie Scholl for the summer semester 1942. She entered by hand the information on this form, such as the names of the faculty (column 2) of the respective course she took (column 3), the number of course units (*Wochenstundenzahl*, ‘number of hours per week spent in class;’ column 4), and the tuition fee paid (column 5). The first course listed is von Frisch’s *Allgemeine Zoologie* (General Zoology), bearing 3 credit hours. She paid a tuition fee of 9 Reichsmark for this course. Courtesy: UAM

Our search in the Archive of the University of Munich revealed that not only Sophie Scholl but also other members of the White Rose group were enrolled in courses of Karl von Frisch.^23^ During the summer semester in 1942—her first semester at the University of Munich—Sophie Scholl took von Frisch’s General Zoology lecture class^24^ (Fig. 4). As a second biology course, she took General Botany, taught by Friedrich Carl von Faber.^25^ Notably, besides two other non-science courses, she was enrolled in two courses (‘Tone and Music Psychology’ and ‘Leibniz^26^ and his Times’) by Kurt Huber, the only professorial member of the White Rose.

In her biography, Barbara Beuys mentions that Sophie Scholl also took at least one course by Karl von Frisch during the winter semester of 1942/1943^27^:

*Sophie Scholl was enrolled during the winter semester in lecture courses of the biologist Karl von Frisch, the physicist Walther Gerlach, the mathematician Georg Faber, the philosopher Kurt Huber, and the archeologist Ernst Buschor*. (Beuys 2021, p. 391)

Since Hans Scholl, Christoph Probst, Alexander Schmorell, and Willi Graf were medical students, and courses in zoology and anatomy were compulsory for the study of human medicine at the University of Munich (Greilinger 2006), we also searched the Archive of the University of Munich for evidence of their registration in any of von Frisch’s classes. These documents^28^ indicate that during the summer semester of 1939 Hans Scholl and Christoph Probst took either von Frisch’s Zoology Lecture Course or the Zoological Lab Course for Medical Students, which he taught jointly with Werner Jacobs.^29^ During the third trimester^30^ of 1939, Hans Scholl, was enrolled in the Comparative Anatomy of Vertebrates Lab Course, which was probably taught by von Frisch,^31^ whereas Christoph Probst and Alexander Schmorell had registered during this term for von Frisch’s Zoology II (Comparative Anatomy of Vertebrates) Lecture Course.^32^ We could not find evidence that Willi Graf read any of von Frisch’s courses. Since he transferred to the University of Munich after he had studied four semesters at the University of Bonn, it is possible that he received transfer credit for equivalent courses taken at the latter institution.

### “Zoology is extremely interesting”

Several lines of evidence suggest that at least the Scholl siblings deeply enjoyed Karl von Frisch’s classes. Yet, in the case of Sophie Scholl the evidence is only indirect. This is rather surprising, given that she was the only biology major among the four who took his classes. Several years earlier, when she had just turned 17 and was still a student at the *Oberrealschule* (high school) in Ulm, she had expressed her enthusiasm for biology in a letter to her sister Inge on July 8, 1938:

*The biology class is great. I have already dissected a bovine eye. I also exposed, so neatly, the internal parts, intestines, organs, etc. of a fish. The brain and the head as well. The entire outer wall was folded back, and then everything inside became accessible. It was so orderly and meaningful and tightly packed. And imagine: the heart was still moving regularly*—*up and down and up and down and up and down. Fish have golden eyes. And a spherical, beautiful lens. Extremely nice animals. I felt so sorry for them*. (Jens 2021, pp. 154–155)

Sophie Scholl was a prolific letter writer, as documented by the high volume of her (published) correspondence between 1937 and 1943 (Hartnagel 2008; Jens 2021). Yet, in none of the letters written after her enrollment at the University of Munich does she mention any classes she took. At first, one might suspect that letters in which she perhaps wrote about her courses are missing. Indeed, most of the letters between March 1941 and February 1943 to her fiancée Fritz Hartnagel got lost in Stalingrad, except those that could not be delivered due to the events of the war and were, thus, returned to her (Hartnagel 2008, p. 11).

However, this explanation is rather unlikely. On February 23, 1943,^33^ Fritz Hartnagel wrote from Lemberg,^34^ where he was deployed as an officer of the German army:

*By the way, you have never written anything about your lecture courses, although for sure they keep you busy for most of the day*. (Hartnagel 2008, p. 457)

Thomas Hartnagel, son of Fritz Hartnagel and editor of the correspondence between Sophie Scholl and Fritz Hartnagel, commented on Sophie Scholl’s priorities by mentioning that Elisabeth Hartnagel,^35^ who had visited Sophie Scholl twice in Munich for several days, was left with the impression that her studies, aside from attending Kurt Huber’s lectures, were of hardly any importance to her. Furthermore, a friend of Sophie Scholl from Ulm, who also visited her in Munich, had to promise in a letter that he would not tell anyone, particularly not her parents, what she really did in Munich (Hartnagel 2008, pp. 458–459).

The only document that indicates how much Sophie Scholl enjoyed Karl von Frisch’s classes is a letter that Inge Scholl^36^ wrote to Karl von Frisch on May 13, 1952. In 1946, she had founded the *Volkshochschule*^37^ in Ulm, of which she was managing director until 1974. In this capacity, she organized a zoology lecture series of distinguished scholars. In her letter, she invited von Frisch to give a lecture. After two introductory sentences, she wrote:

*My two siblings attended your lectures with great pleasure in 1942.*^38^* They told me about your experiments.*^39^

While this letter of Inge Scholl is the only documented evidence that Sophie Scholl enjoyed Karl von Frisch’s course, there is (besides this letter) some direct evidence that Hans Scholl liked the zoology classes. A few days after he had enrolled at the University of Munich, on April 17, 1939, he wrote in a letter to his parents:

*Of all the mandatory lecture courses, the nicest has been botany so far. However, also zoology is extremely interesting. Friday afternoon, I will, most likely, register for the zoology lab (3 units).*^40^

### Was Karl von Frisch aware of the actions of White Rose and the fate of its members?

Was Karl von Frisch aware of the activities, as well as the arrests, trials, and executions of members of the White Rose, including Hans Scholl, Christoph Probst, Alexander Schmorell, and Sophie Scholl (all of whom had taken classes with him), and his professorial colleague Kurt Huber? And, after World War II, did he ever comment on the anti-Nazi resistance at his own university?

Despite an intensive search in the Archive of the University of Munich and in the collection of documents in von Frisch’s estate at the *Bayerische Staatsbibliothek*, we have not found any evidence indicating that he ever mentioned the activities of the White Rose or any of its members.

One might argue that his presumed abstention from commenting on the White Rose during World War II was due to him being unaware of their actions as well as their arrests, indictments, and executions during World War II; and, after World War II, when members of the White Rose became iconic figures symbolizing anti-Nazi resistance (although they, like other individuals and groups, had failed to mobilize mass political opposition), he did not realize that at least four of them had taken classes with him.

However, besides von Frisch’s correspondence with Inge Scholl, several historical facts strongly argue against such a notion. During the war, it is extremely unlikely that any student or faculty member of the University of Munich did not become aware of at least some of the actions of the White Rose and the events that followed the arrest of Hans and Sophie Scholl on February 18, 1943.

First, between June 1942 and February 1943, members of the White Rose scattered by hand thousands of copies of the six leaflets on campus and at public places in Munich. Leaflets were also mailed to select individuals, including professors and academic staff of the University, whom they hoped to win over to their cause. Most significantly, documented evidence proves that at least two zoologists—Werner Jacobs and Hans Krieg^41^—received leaflets mailed to their residential addresses by the White Rose. Jacobs, von Frisch’s right hand, got the second and third leaflet, postmarked July 1, 1942 and July 6, 1942, respectively. Hans Krieg was recipient of only the second leaflet, postmarked July 21,1942. Each of them handed over their copies to the Gestapo.^42^ It is unknown whether Jacobs and Krieg discussed the leaflets with other members of the Zoological Institute, including von Frisch. It remains also elusive whether other zoologists, including von Frisch, received leaflets from the White Rose but did not report them to the Gestapo.

Second, in early 1943, Alexander Schmorell, Hans Scholl, and Willi Graft began a graffiti campaign, painting slogans like *Freiheit* (Freedom) and *Nieder mit Hitler* (Down with Hitler) on buildings throughout Munich, including the University.

Third, when Hans Scholl, Sophie Scholl, and Christoph Probst were found guilty of treason, condemned to death, and executed on February 22, 1943, a press release by the Bavarian State Government announced the news the same evening, which were published on the next day in several newspapers, including the local edition of the *Völkischer Beobachter* as well as the *Münchner Neueste Nachrichten* and the *Münchener Zeitung*.

Fourth, on the evening of the execution, following an invitation from the University’s leadership, 3000–4000 students rallied to express their support for the *Führer* and the Nazi movement, and to condemn the actions of the convicted members of the White Rose.

**Fig. 5:**
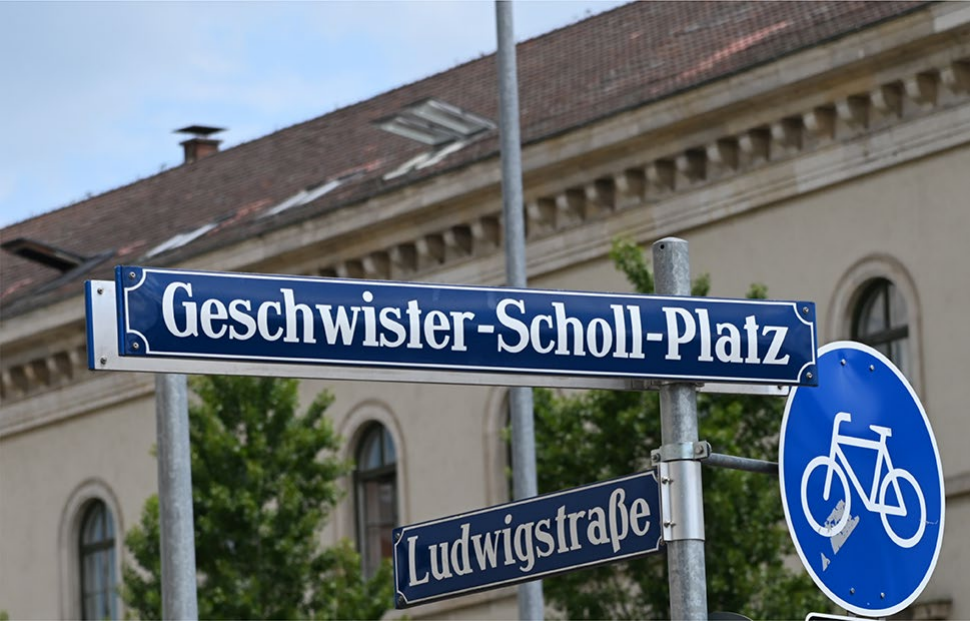
*Geschwister-Scholl-Platz* (Scholl Sibling Square), named after Hans and Sophie Scholl and located in front of the main building of the University of Munich. Photograph by Günther K.H. Zupanc

**Fig. 6:**
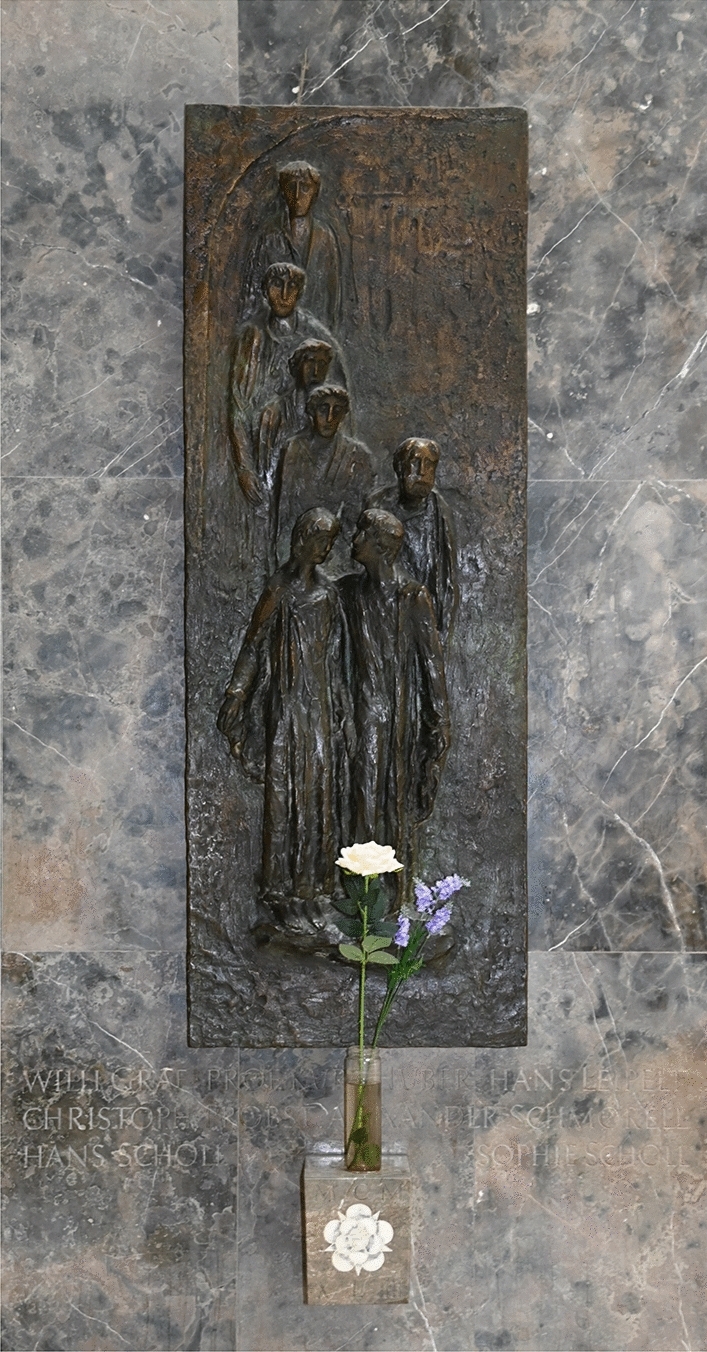
Bronze relief commemorating the seven executed members of the White Rose—Hans Scholl, Sophie Scholl, Willi Graf, Hans Leipelt, Christoph Probst, Alexander Schmorell, and Kurt Huber. It is mounted on the western side in the atrium of the main building in which Hans and Sophie Scholl were arrested. Created by the German sculptor Lothar Dietz, this piece of artwork was unveiled in 1958. Photograph by Günther K.H. Zupanc

After the war, the first public commemorative event honoring the members of the White Rose took place as early as on November 4, 1945,^43^ just six months after the unconditional surrender of the German Third Reich. The next year, on September 9, 1946, the forecourt of the University’s main building and the court opposite were named *Geschwister-Scholl-Platz* (Scholl Sibling Square) (Fig. 5) and *Prof.-Huber-Platz* (Professor Huber Square), respectively. On November 2, 1946, a plaque made of Jura marble^44^ was unveiled. Its Latin inscription commemorates the seven members of the White Rose, “who had to die an inhumane death for upholding the values of humanity.” A bronze relief commemorating the seven members of the White Rose, mounted on the western side in the atrium of the main building, was unveiled in 1958 (Fig. 6).^45^ Over these years, the White Rose has become *the* symbol of non-violent resistance during the Nazi era, and several members of the group, in particular the Scholl siblings,^46^ evolved as national heroes in both East and West Germany.

**Fig. 7:**
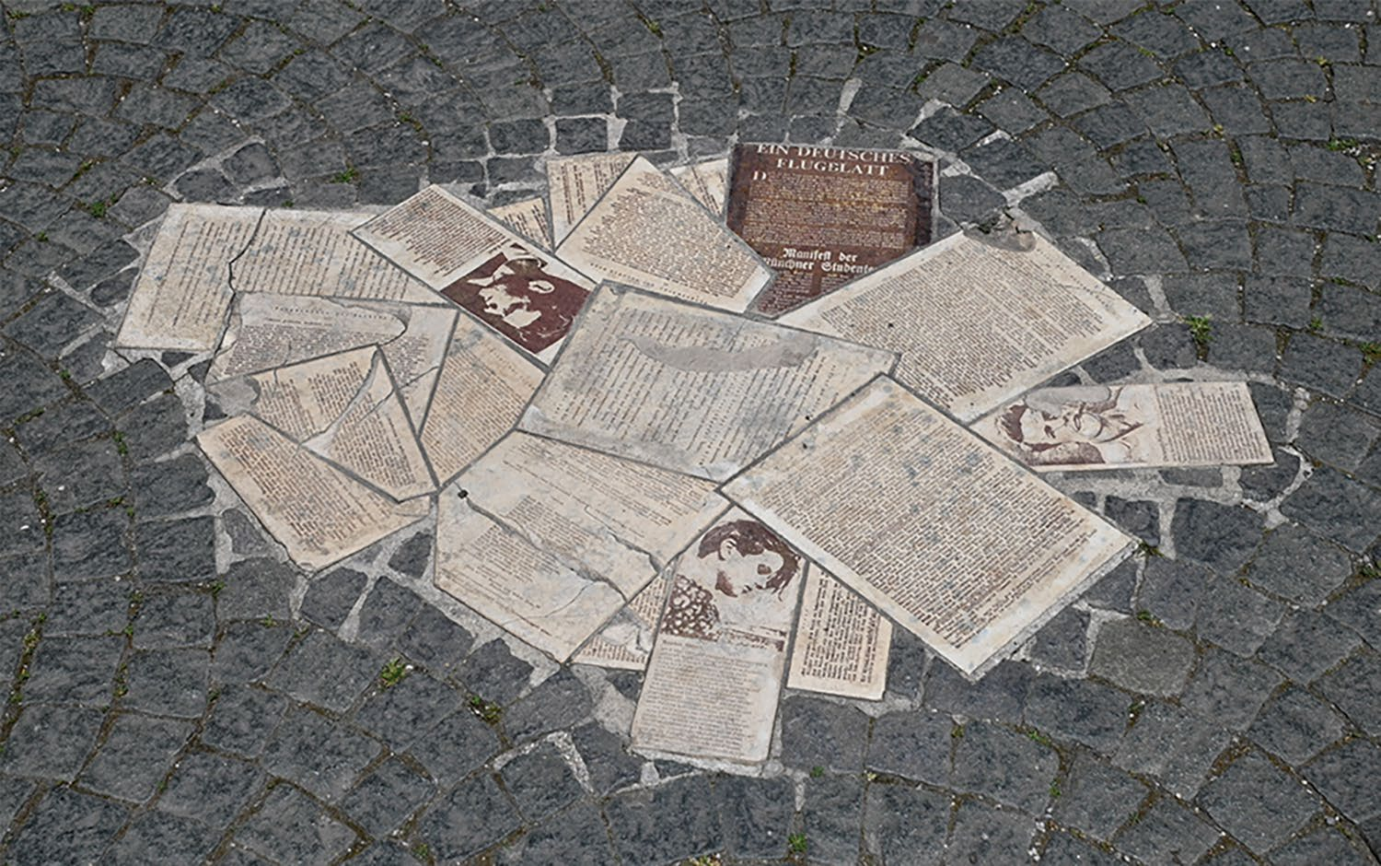
Pavement memorial to the White Rose. Embedded in the cobblestone pavement in front of the main entrance of the university building, the ceramic tablets by artist Robert Schmidt-Matt depict photographs and biosketches of Hans Scholl, Sophie Scholl, and Christoph Probst, as well as leaflets of the White Rose. Photograph by Günther K.H. Zupanc

Thus, we consider it to be virtually impossible that, both during and after the war, Karl von Frisch had not learned about the actions of the White Rose group and the events that followed the arrest of Hans and Sophie Scholl. However, did he know that four of its members, including Hans and Sophie, were students in his classes?

To weigh in on this question, it is helpful to look at the number of students at that time, both in the biological-discipline programs (botany and zoology) and in the courses taught by Karl von Frisch. During the summer semester of 1942—Sophie Scholl’s first semester—a total of 3788 students were enrolled at the University of Munich.^47^ Out of them, 1877 were medical students and 389 were students in the College of Science. Of the latter, 115 students were in their first semester. Based on the fact that botany and zoology students comprised 15–30% of the science students at the time of graduation,^48^ we estimate that Sophie Scholl was one of 17–35 students who commenced their studies in these two disciplines in the summer semester of 1942. This number is notably low, making it likely that professors, including von Frisch, knew these students in person.

Our analysis of students who were enrolled in Karl von Frisch’s lecture courses indicated large numbers. For example, the General Zoology course in the summer semester of 1942 was taken by 451 students, including Sophie Scholl. However, this situation was different for lab courses. For example, analysis of the course registration cards for the 3rd trimester of 1939 revealed a total of 69 students in the Comparative Anatomy of Vertebrates lab class, including Hans Scholl. Considering the reputation of von Frisch and Jacobs as highly engaged instructors, we consider it to be likely that some personal interaction took place between Scholl on the one side and von Frisch on the other.

Whereas the student numbers make it likely that Karl von Frisch knew members of the White Rose personally, there is documented evidence that he was reminded of them after the war. Inge Scholl, in a letter dated May 13, 1952, to Karl von Frisch mentioned that Hans and Sophie Scholl had attended his lectures “with great pleasure” (see section ‘“Zoology is extremely interesting”,’ above). In a reply dated May 15, 1952, von Fisch declined the invitation to give a presentation at the *Ulmer Volkshochschule*, while he did not address Scholl’s remarks on her siblings. Over the following five years, Inge Scholl sent another eight letters to von Frisch in which she repeated her invitation. His replies are documented in three letters preserved in the BSB archives. According to the correspondence, he finally gave a lecture at the *Ulmer Volkshochschule* on December 5, 1957.

Notably, however, in a letter of July 19, 1956, Inge Scholl wrote that she would be in Munich between July 23rd and July 27th, and that then she would call him to arrange, if possible, an in-person meeting. In what probably was her next letter, dated October 10, 1956, she referred to a *persönliche Unterredung* (conversation) that took place in July of that year. However, it remains unclear whether they had talked over the phone or during an in-person meeting in Munich. Thus, based on the documented exchange of 13 letters between 1952 and 1957, we assume that they met at least once (when Karl von Frisch gave his lecture in Ulm in 1957). We also have evidence that they talked to each other on at least one other occasion, in 1956. It is unknown whether, during the two conversations that presumably took place, the White Rose was mentioned. Nevertheless, this was likely the case, given that Inge Scholl published her book *Die Weiße Rose* around the same time, in 1955 (Scholl 2021), and that, since the execution of her siblings until her death in 1998, she devoted her life to telling the story of her siblings.

## The (a)political nature of Karl von Frisch during the Nazi era

### “I kept up the spirit of doing research … by ignoring what happened around me”

At about the same time when Inge Scholl published the book that established members of the White Rose as iconic figures of the anti-Nazi resistance, Karl von Frisch wrote his memoirs (von Frisch 1957). In the entire book, he comments on political resistance only once, when he briefly mentions the role that universities and professors played during early years of the Nazi era:

*If there had been consensus among universities, they may perhaps have succeeded in fighting back. However, many professors applauded the new movement, some because they wanted to avoid confrontation, others because they were ardent Nazis. Nonetheless, soon it had become clear that any significant resistance would result in self-destruction*. (von Frisch 1957, p. 113)

This quote provides some important insight into von Frisch’s political views—not only did he regard political resistance during the Nazi era as an act of self-destruction, but he also made any attempt to stay out of politics. The latter point is articulated even clearer in the following text in his memoirs, in which he outlines his strategy to maintain a high level of scholarly productivity during times of political oppression:

*I kept up the spirit of doing research by immersing myself into work and, as much as possible, by ignoring what happened around me, which was beyond our control*. (von Frisch 1957, p. 120)

When major parts of Munich, including the house of his family in the Harlaching municipality of Munich and the Zoological Institute of the University of Munich, were completely destroyed or severely damaged by allied bombs, a highly effective way for Karl von Frisch to “ignore what happened around him” was to relocate his residency and a significant amount of his research operation to his country estate in Brunnwinkl at Lake Wolfgang, Austria (for a historical account of Karl von Frisch’s family estate and for its importance for his research see von Frisch 1980). Shortly after the war, in 1946, he even retired from his *Ordinarius* position in Munich and accepted an offer to join the faculty of the University of Graz in the Southern Austrian province of Styria. In contrast to Munich, there had been relatively little destruction during the war in Graz, so that Karl von Frisch could invest his efforts into continued research, instead of rebuilding the Munich Zoological Institute (a task that was left largely to his assistant Ruth Beutler; see Zupanc 2024 in this issue). Perhaps equally important, von Frisch perceived this move as a strategic decision to avoid being disrupted by memories of the past:

*Even just the thought of Munich evoked painful memories and feelings associated with what we had witnessed over the last decade. I was hopeful that a change of location would provide me with freedom from worry and anxiety*—*the kind of mental state that science needs to flourish*. (von Frisch 1957, p. 131)

The apolitical nature of Karl von Frisch that transpires from the above statements aligns with the recollection of one of his grandchildren, Julian von Frisch.^49^ During an interview,^50^ he told us that he does not remember his grandfather ever talking about politics in general or mentioning the White Rose in particular, and that he used to ignore whatever might have negatively interfered with his work.^51^

### The political strategy of Karl von Frisch during the Nazi era

Karl von Frisch’s declared efforts to stay out of politics did not prevent him from utilizing politically charged topics when it benefitted his own interests.^52^ This is particularly true for ‘race,’ the issue that defined the political agenda, and ultimately the atrocities, of the Nazis more than any other. In 1936, he published *Du und das Leben: Eine moderne Biologie für Jedermann* (Title of the English editions: Man and the Living World) (von Frisch 1936). In this highly successful introductory biology textbook, he covered not only plant and animal breeding but also *Rassenhygiene* (racial hygiene, eugenics^53^) in humans as one of its chief applications. Notably, the extent to which this topic was covered, and its content, differ between the two editions of the book. The original edition was published by Ullstein Verlag, whereas other editions (e.g., von Frisch 1940) appeared in the Deutscher Verlag during the Third Reich. The latter was the successor of the Jewish Ullstein Verlag, which was aryanized^54^ in 1934 and renamed Deutscher Verlag in 1937. It was affiliated with the Franz Eher Nachfolger publishing house of the Nazi Party. At least one of the Deutscher Verlag editions received financial support through the *Dr.-Goebbels-Spende für die Deutsche Wehrmacht*,^55^ a fund named after Josef Goebbels^56^ and intended to provide the *Deutsche Wehrmacht* (German armed forces) with books for entertainment and education. The Ullstein and Deutscher Verlag editions differ at the end of the book. Whereas the Ullstein edition contains, as its final part, a one-page section on eugenics, the coverage of this topic is expanded to eight pages in the Deutscher Verlag edition.

A section immediately preceding the coverage of eugenics is titled *Zukunftssorgen* (Worries about the Future). In this text part, Karl von Frisch laments that unhealthy phenotypic deviations, which are weeded out “mercilessly” in indigenous people and wild animals but persist in highly developed cultures, due to humanitarian sentiment and medical support. According to his diagnosis, the abolishment of benevolent selection by giving preference to inferior people in modern societies, and the unrestricted reproduction of these individuals, result in a relative increase of people with heritable mental illnesses or physical malformations, “who, by receiving support from society, live at the expense of hardworking people” (von Frisch 1940, p. 350).

In the two sections of the Deutscher Verlag edition that follow the Worries-about-the-Future section, Karl von Frisch discusses how this decline of human races, as he sees it, can be prevented. The proposed solution is based on his assumption that races differ not only in morphological traits but also in their mental potential. ‘Crossbreeding’ of these races, as he argues, produces numerous phenotypes, among which many are *unerwünscht* (undesirable) and should, therefore, be prohibited. “Keeping races pure is the basic idea of eugenics, which has been implemented in a powerful way through the legislation^57^ of the German Reich … in our society, the *Rassenpolitische Amt*^58^ serves as a consultant to the government concerning this and other issues” (von Frisch 1940, pp. 352/353).

To counter social degeneration and decline of human civilization, allegedly caused by the lack of natural selection, von Frisch advocates for forcible sterilization. Given his sympathetic view of such measures as a legitimate and necessary approach to enforce racial hygiene, it is not surprising that in an article that Karl von Frisch published in the high-profile weekly Nazi newspaper *Das Reich*^59^ in February 1941 (von Frisch 1941), he praises the German Reich in its effort to utilize the work of contemporary genetics, “in the broadest form”, for the benefit of the *Volk* (nation, race).

## The political naivety of Karl von Frisch

Whereas Karl von Frisch’s writings during the Third Reich document how he used his demonstration of support of some of the political agendas of the Nazis as a strategy to achieve his research goals, it appears that at least some of his actions were guided by political naivety. This feature, which emerges as a characteristic trait of him, is illustrated by four examples detailed below.

### In defense of Franz Wirz, the “right-minded man”

The first example relates to the threat of his forced retirement during the Third Reich. After the *Gesetz zur Wiederherstellung des Berufsbeamtentums* (Law for the Restoration of the Professional Civil Service) had been signed into law on April 7, 1933, civil servants, including university professors, had to provide proof of their Aryan descent and give assurance that they would support the national state at any time. In the corresponding questionnaire, von Frisch had indicated catholic faith of his parents and grandparents. The university acknowledged Aryan descent in an official list issued July 18, 1933.^60^

However, in 1936, a re-review of von Frisch’s claim of Aryan descent was initiated after two assistants of the Zoological Institute had testified that at least one of his grandparents was a Jew (for a detailed account see Zupanc 2024 in this issue). This triggered inquiries by several authorities of the Bavarian state government in Munich and the Reich government in Berlin, which, in 1941, led to the notification by the Reich Minister of Science, Education and National Culture of their intention to retire him early. Karl von Frisch fought this decision by petitioning to reverse the intended forced retirement and by demonstrating his loyalty to the Reich, for example by publishing an article in the high-profile Nazi newspaper *Das Reich* (von Frisch 1941), as mentioned above.

At the same time, he rallied support by several colleagues. Although some details remain elusive, the decisive help came from a Nazi official, Franz Wirz (1889–1969), who served in various leadership positions, including head of the *Hauptamt für Volksgesundheit der NSDAP-Reichsleitung* (Head Office for People’s Health at the Headquarter of the Nazi Party). In this function, he had, among other assignments, the task of combatting nosemosis, an infection of adult honeybees (*Apis mellifera*) with the microsporidium *Nosema apis*. Addressing this issue had become a topic of national interest when, at the beginning of the 1940s, beekeepers in Germany and other countries suffered heavy losses totaling hundreds of thousands of bee colonies due to nosemosis. This resulted in a severe reduction in the production not only of honey but also of food crops dependent on insect pollination. After Wirz’s intervention, von Frisch was commissioned by the Reich Ministry of Food and Agriculture to coordinate the scientific efforts for combatting nosemosis. To enable von Frisch to carry out this project, the Reich Ministry of Science, Education, and National Culture agreed to delay his retirement until the time after the war.

Like many other Nazi officials after the war, Franz Wirz was dismissed from his position by the Allied Military Government and held at a detention facility from December 1945 to November 1947. On January 10, 1946, Karl von Frisch wrote a letter to the commanding officer of the Counterintelligence Corps (a World War II and early Cold War intelligence agency within the United States Army) Detachment in Munich.^61^

*I have learned that Professor F. Wirz was arrested end of December. I do not know what charges have been brought against him. However, I would like to share some information with you in his defense.*

Then, von Frisch states how Wirz’s intervention on his behalf was critical in delaying his forced retirement until the end of the war. He finished the letter by commenting on Wirz’s character:

*… he deeply cares about supporting research. I had the impression that not only in regard of the latter and the Jewish problem, but also otherwise Professor Wirz was a right-minded man who used his power to vehemently counter the madness of the Nazi party in quite a few instances.*

Whereas von Frisch’s testimony of the support he received from Franz Wirz appears to be correct, his portrait of Wirz as a “right-minded man who used his power to vehemently counter the madness of the Nazi party” is, at the very least, incomplete. Wirz had studied medicine at the University of Munich and received his M.D. degree in 1916 (for biographical details see Grüttner 2004). After World War I, he worked as a dermatologist at the university hospital in Munich. In 1927, he was appointed to a non-tenure-track professorship at the University of Munich.

Immediately after Hitler seized power in 1933, Wirz became a member of the Nazi party. He was one of the masterminds of a massive denunciation campaign against Leo von Zumbusch (Böhm 1995). Von Zumbusch (1874–1940) was not only Wirz’s line manager at the university hospital but also rector of the University of Munich from 1933 to 1934.^62^ (Although von Zumbusch was the first rector after the Nazis had seized power, he had still been elected, in 1932, during the Weimar Republic.) As a result of Wirz’s denunciation campaign, von Zumbusch was forced to retire early in 1935 under the Law for the Restoration of the Professional Civil Service because of political reasons. The accusations included, among others, his refusal to give the Nazi salute and derogatory comments that he had made about Hitler.

Since von Zumbusch was a prominent professor at the University of Munich, it is likely that Karl von Frisch, like other faculty colleagues, was aware of the circumstances of his dismissal. This is particularly the case because von Frisch and von Zumbusch must have known each other well. In 1906, von Zumbusch had married his first wife, the Viennese artist Nora Exner (1879–1915), the daughter of Adolf Exner (1841–1894).^63^ The latter was the brother of Karl von Frisch’s mother, Marie von Frisch (née Exner) (1844–1925).^64^ In other words, Nora von Zumbusch (née Exner) was Karl von Frisch’s cousin.

### Konrad Lorenz, the army psychologist and advocate of racial hygiene: “It would be a pity if his research were hampered by untrue rumors”

The second example illustrating Karl von Frisch’s political naivety relates to a letter that he wrote in Minneapolis (Minnesota) on May 14, 1949, while he was on a three-month lecture tour through the United States. This letter was addressed to Niko Tinbergen. In its introductory notes, von Frisch expressed his hope to receive from him some information about one of Tinbergen’s friends, Konrad Lorenz.

*Lorenz would like to acquire funding from the Rockefeller Foundation for his research. However, there is wide-held suspicion here [in the United States] that he was an active Nazi. I am not aware of that, and I have a hard time believing it. Probably, it is true that he was a member of the [Nazi] party.*^65^* Rumors are that he was a* Heerespsychologe* (army psychologist), which people here interpret as involvement in Nazi activities. I don’t know whether there is any particular meaning to these activities … I would appreciate … if you could provide me with more information. It would, for sure, be a pity if Lorenz’s research were hampered by untrue rumors.*^66^

In his reply, Niko Tinbergen confirmed that they were friends and that he knew Lorenz very well.

*After his release as a prisoner of war from a Russian internment camp, we resumed our correspondence. Of course, I asked him about the rumors regarding his sympathies for the Nazi ideology.*^67^* I think, he told the truth when he responded that initially he felt positive about the Nazis, but soon he realized his mistake. I do not know anything about his activities as a* Heerespsychologe*. As far as I know, he served as a medical doctor … I cannot tell much more because, after we were in contact again, we agreed not to write about politics.*^68^

Lorenz himself never mentioned anything about such a role in public. In autobiographical pieces, he described two functions in which he served in the Wehrmacht—as a *Kradschütze* (a member of a motorized, lightly armed, and highly mobile infantry unit) and a medical doctor. Only three years after his death in 1989, first indications surfaced about Lorenz’s involvement as an army psychologist in a comparative ‘race psychology’ study on people of mixed German-Polish background, which was conducted in Posen (Polish Poznań) in occupied Poland under the leadership of Rudolf Hippius^69^ in 1942. This research, best known as the ‘Posen study,’ was based on psychological tests and observations of 877 subjects carried out by a group of interviewers. This team included Konrad Lorenz, who is listed as a ‘volunteer’ and probably spent several hours every week interviewing subjects, on top of his main duties as a military psychiatrist and neurologist.

Although Lorenz did not co-author the 416-pages long monograph of the Posen Study (Hippius et al. 1943), its main conclusions aligned well with the core message that he conveyed widely in publications and lectures during the Third Reich, including a comprehensive, 80-pages long paper entitled *Durch Domestikation verursachte Störungen arteigenen Verhaltens* (Defects of Species-Specific Behavior Caused by Domestication), which had appeared three years earlier (Lorenz 1940). In this highly speculative article, Lorenz first describes how domestication of animals, through selection by the breeder of mutants whose fitness is reduced under natural conditions, results in abnormal changes in instinctive behaviors and defects in the selectivity of an animal’s response to releasers, particularly in the context of reproductive behavior, pair bonding, and brood care. Lorenz postulates that these defects are primarily caused by “complete absence” of what leads under natural conditions, through selection, to the eradication of such behavioral mutants. Additionally, as he speculates, an increase in the frequency at which the underlying mutations occur may also contribute to the observed behavioral defects.

In the second major part of his treatise, Lorenz postulates that behavioral defects that are homologous to those in domesticated animals occur in humans living in big cities.

*From a racial hygiene point of view, such defects in the social behavior of civilized humans are the most dangerous and malicious ones … and, in all likelihood, are caused by the same factors.*^70^

He, then proposes two remedial strategies.

*If there are factors that increase the likelihood of mutations resulting in such defects, then their identification and elimination would be the prime duty of the racial hygienist (‘Rassenpfleger’) because the occurrence of humans with such defects in species-specific social behaviors is most detrimental to nation and race (‘Volk und Rasse’) … On the other hand, if it will be shown that the increase of mutants [with behavioral defects] is not caused by an increase in the frequency of mutations but by the absence of natural selection, and an imbalance of the races, then the racial hygienist must ensure an even more stringent eradication (‘Ausmerzung’) of ethically inferior people than done today. Racial hygiene would, thus, have to replace all the selection factors that are at work under natural conditions … even a small defect in social inhibition exhibited by a group makes their carriers, under the condition of life in the city, capable of outsmarting their fully-fit conspecifics (‘vollwertige Artgenossen’) and developing into a parasite for the nation as a whole (‘das Volksganze’) … this phenomenon results … in a situation in which inferior human material (‘minderwertiges Menschenmaterial’) become, due to exactly these inferior properties, able of invading the healthy body of the Volk (‘gesunder Volkskörper’) and ultimately to destroy it.*^71^

Lorenz concludes his wild speculations by praising the achievements of national socialism:

*The notion of race as the foundation of our nation has done an infinite amount of good. The Nordic movement has always been directed against the domestication of humans. Its ideals are those that would be destroyed by the biological consequences of civilization and domestication. It, therefore, fights for a development antagonistic to the direction in which today’s humans of the cities move. To people who think biologically, there is no doubt which of the two ways is the way of evolution, the way ‘up’!*^72^

Although Konrad Lorenz never spells out what he envisages when he demands “eradication of ethically inferior people” as a substitute of natural selection, it is clear that his (largely pseudoscientific) analysis could not only be used as retroactive legitimation of the Nuremberg Laws^73^ and the enforced sterilization of “inferior human material” under the Law for the Prevention of Hereditary Diseased Offspring but also lend ‘scientific’ support for further escalation that started with World War II and culminated in the mass murder of persons with disabilities, as done in the *T4 Aktion*, and people viewed as racially inferior by the Nazis, such a Jews, Roma, and Sinti.

As with the case of Lorenz’s role as an army psychologist during World War II, one would like to know whether Karl von Frisch was aware of Lorenz’s 1940 paper. Unlike Lorenz’s participation as an army psychologist in the Posen Study in 1942, which he managed to keep as a secret throughout his lifetime, the situation is less obvious in the case of his 1940 article. Not only was this article publicly available, but Lorenz was also very active around that time in promoting his paper and its content through lectures and coverage in other journals and newspapers, such as *Der Biologe*, *Neues Wiener Tagblatt*, *Das Reich*, and *Völkischer Beobachter* (Föger and Taschwer 2001). Since the community of the just-emerging discipline of animal psychology (later to become known as ethology) was then very small, and the number of papers published by its representatives was miniscule (compared to today’s measures), it is likely that von Frisch was at least aware of the core thesis of Lorenz’s 1940 paper.

### Karl von Frisch and the Simon Wiesenthal petition letter

The third example that is presented here as illustration of Karl von Frisch’s political naivety is based on a letter^74^ (dated November 2, 1964) he had received from Simon Wiesenthal^75^ of the *Dokumentationszentrum des Bundes jüdischer Verfolgter des Naziregimes* (Documentation Center of the Association of Jewish Victims of the Nazi Regime) in Vienna. In the letter, which in identical form was sent to several hundred members of the intellectual elite in Germany and Austria, Wiesenthal asked the recipient to submit an expression of opinion on the extension of the statute of limitations on Nazi crimes involving murder. At that time, the limitation on prosecuting crimes punishable by life imprisonment was twenty years in Germany and Austria. Due to the juridical deficiencies of the Third Reich, the date of the beginning of the limitation period was set for May 8, 1945 (the day when World War II ended in Europe). As a result, crimes of murder committed during the Nazi era were still punishable only until May 8, 1965 (for a political, legal, and historical review see Monson 1982).

Approximately 90% of the respondents to Wiesenthal’s letter—360 in total—had supported an extension of the statute of limitation. These responses were presented to the German Government on January 28, 1965, and to the Austrian Government on February 3, 1965. After a prolonged debate by the members of the German *Bundestag* (Parliament), a (temporary) solution was adopted by the House on March 25, 1965, by moving the beginning of the twenty-year tolling period for murder to December 31, 1949,^76^ thereby giving the authorities more time to prosecute crimes committed by Nazi Germany.

Compared to many of the other respondents, and to the letters that Karl von Frisch usually wrote to colleagues in important matters, his response to Wiesenthal’s request was very short (seven typewritten lines) and somewhat superficial. He expressed his objection of any statute of limitations of crimes committed during the Nazi era, and he also suggested that changes in the existing law be applicable only to *wirkliche Verbrecher* (true criminals) but not to *kleine Mitläufer* (followers or accomplices). These comments indicate von Frisch’s limited familiarity with the issue, although the statute of limitations of murder crimes during the Third Reich was perhaps the most contentious issue debated by politicians and the public in 1964. First, in contrast to von Frisch’s proposal, complete abolishment of the statute of limitations was not considered at that time. Second, it is unclear what crimes he associated with followers or accomplices. For both non-homicidal crimes and for manslaughter, the deadlines had already passed (May 8, 1955, and May 8, 1960, respectively). Moreover, by 1964 it had become increasingly clear that the ‘follower’ argument was used by many Nazi criminals to escape adequate punishment—they claimed they had acted ‘only’ as followers or accomplices, even though they had been involved in murder (as defined by the penal code) of numerous people.

### Karl von Frisch’s endorsement of Werner Jacobs, member of the Nazi party and the SA

The fourth and final example used here to illustrate the political naivety of Karl von Frisch concerns Werner Jacobs. As mentioned above (see footnote 29), he was an active Nazi during the Third Reich, which is underscored by his membership in eight Nazi organizations, including the SA, in which he served during the entire Nazi era. On January 6, 1946, Jacobs was suspended from his tenured *Konservator* position, but reinstated on August 8, 1948.

Karl von Frisch had retired from his *Ordinarius* position at the University of Munich in 1946 and accepted the offer to join as *Ordinarius* the University of Graz in Austria. However, despite his official absence from Munich over the following few years, he continued to pull the strings in the search for his successor. He was regularly informed about any significant development in the search process through correspondence with his close confident, the interim *Ordinarius* Ruth Beutler (for further details see Zupanc 2024 in this issue). After the first search had ended unsuccessfully, a new list of three candidates was established. On top of this list was Erich von Holst, followed by Werner Jacobs and Anton Koch. In a letter to the Bavarian State Ministry for Education and Culture, von Frisch endorsed both von Holst and Jacobs. (After von Holst declined the offer, in a surprising move of the University of Munich von Frisch was offered the vacant position; he accepted this offer and returned to Munich in 1950.)

While it seems highly unlikely that Karl von Frisch did not know about Jacobs’ active support of the Nazi movement during the Third Reich, it is virtually impossible that he was not aware of the reason for Jacobs’s two-and-a-half-year suspension from his university position. Details of his suspension, including his membership in eight Nazi organizations, are clearly noted in his employment file of the university. Thus, von Frisch’s endorsement of Jacobs as his successor is perplexing. It seems that either the ‘brown’ past of Jacobs was not an issue at all for von Frisch, or Jacobs scientific merits took precedence over any concerns about his support of the Nazi regime.

## Epilogue

At first sight, it is puzzling that Karl von Frisch seemed to have completely ignored the events that unfolded following the actions taken by the White Rose resistance group in front of his eyes in 1942 and 1943; and, after World War II, when key actors of these events emerged as national heroes and important figures in Germany’s history. Did he not remember that four of them were students in his classes, even when he was reminded of this fact by their main biographer?

Moreover, as a quarter-Jew, he himself was a victim of the totalitarian Nazi regime, and he knew from his own experience that without the help of others it was virtually impossible to counter the oppressive measures of the Reich government. However, we have not found any indication that he supported opposition to the Nazi regime, or that he even just expressed his sympathy for such activity.

Only a few years earlier, it was exactly this kind of support that von Frisch had provided to help victims of political persecution. Some indication of his help is even documented in an official letter dated May 15, 1936, that was written to the Reich Ministry of Science, Education and National Culture in Berlin^77^ by the leaders of the *Dozentenbund* and *Dozentenschaft* of the University of Munich, Wilhelm Führer und Ernst Bergdolt, respectively.^78^ In this eight-pages-long letter, they denounced him as a *Judenstämmling* (a person with Jewish ancestry) and for his favoritism toward Jewish scientists. Among the latter, they listed Otto Egon Löwenstein,^79^ who was employed at the Zoological Institute at the time when the Nazis seized power; Curt Stern,^80^ whom von Frisch had considered for a tenured *Konservator* position at his institute shortly before Hitler was appointed chancellor of Germany; and Dora Ilse,^81^ who had worked for several years as a *private wissenschaftliche Hilfskraft* under von Frisch. Four years later, in 1940, Karl von Frisch went even as far as initiating the release of a Polish colleague, Roman J. Wojtusiak, from the Dachau concentration camp.^82^

Why, then, did Karl von Frisch never express any sympathy for the White Rose? Did he have in mind the fate of its members when he wrote in his autobiography that “resistance leads to self-destruction”?

While it may be true that he ignored what happened around him to keep up the spirit of doing research (as he phrased it), it also appears that, in the early 1940s, he changed his strategy of how to cope with the Nazi regime. This change may have been triggered by the dramatic worsening of the political situation around 1942/1943, which was catalyzed in large by Germany’s defeat in the Battle of Stalingrad. The worsening of the political situation is reflected, among many other measures, by an enormous surge in the number of executions, most of which were related to alleged political crimes. Out of the estimated 16,000 civilians that were executed during the twelve-and-a-half years of the Third Reich, 5,336 were executed in 1943 alone.^83^ The latter number includes the six core members of the White Rose. Considering this worsening of the political reality, Karl von Frisch might have felt it to be necessary not only to abandon his help for victims of the Nazi regime but also to publicly praise some of the ‘achievements’ of the Reich government, particularly in the context of eugenics.

**Fig. 8:**
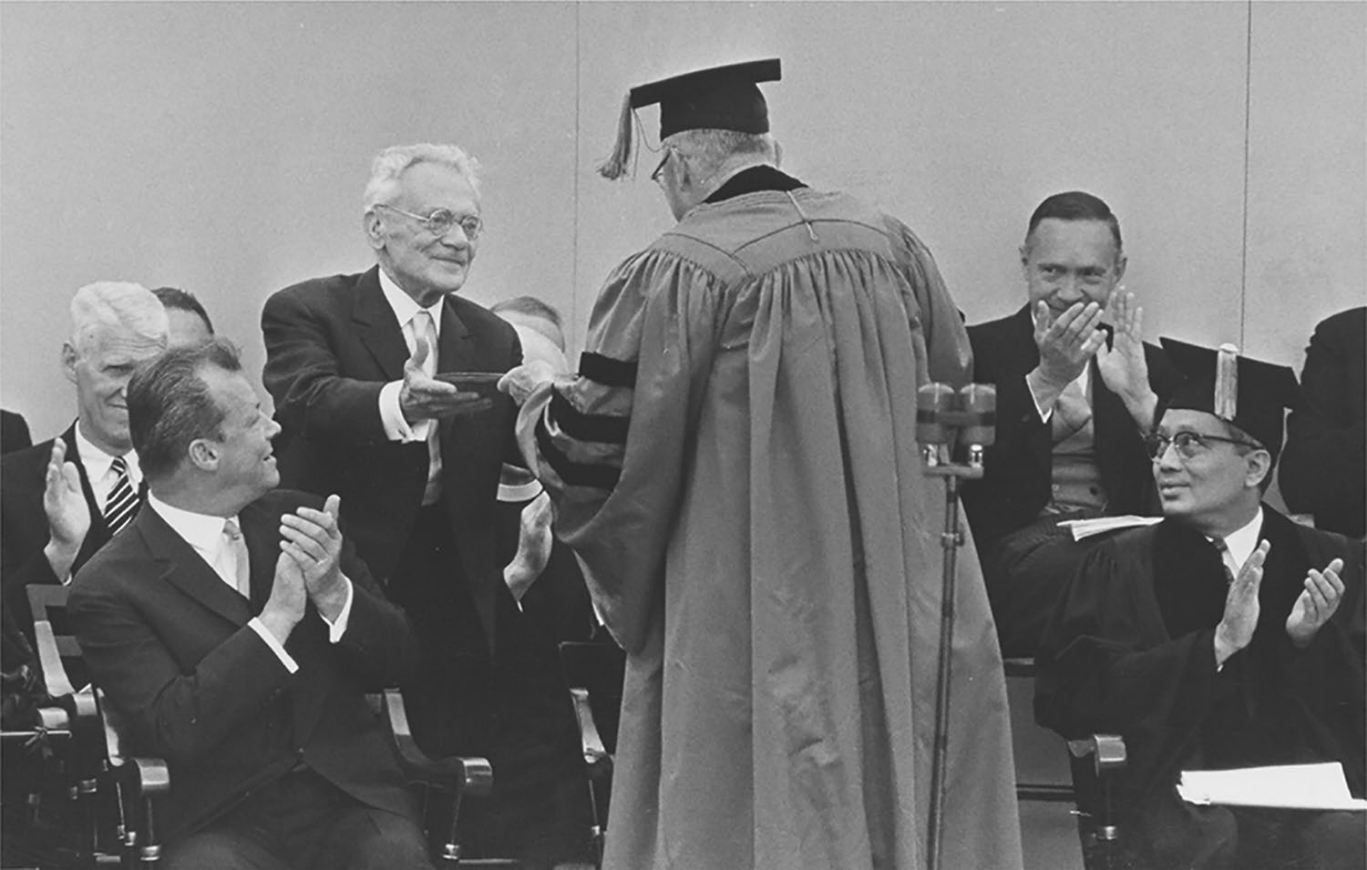
Karl von Frisch receiving an honorary doctorate from J. Hampden Robb, University Marshal, during the 312th commencement of Harvard University in 1963. The honoree applauding in front of von Frisch is Willy Brandt, then Mayor of West Berlin. Brand served as the chancellor of West Germany from 1969 to 1974. He was awarded the Nobel Peace Prize in 1971, two years before von Frisch received a Nobel Prize for Physiology or Medicine. Courtesy: BSB Ana-540, E.I.14

Had he shown any sympathy or even support for opponents of the Nazi regime (including members of the White Rose) during the later years of the war, it is almost certain that, at the bare minimum, he would have lost his position at the University of Munch. On the other hand, had some of the Munich students not shown the world that among all the perpetrators and followers under Hitler there were also different Germans, Germany’s path to becoming a member of the new Europe after World War II might have been far longer and more arduous. The difference in the consequences for the main actors of this historic drama was that Karl von Frisch’s silence, and his arrangements with the Nazi regime, enabled him to keep his position and to carry out some critical pieces of his research. For these achievements, he received numerous accolades, including honorary doctorates from six universities (Fig. 8) and a Nobel Prize for Physiology or Medicine in 1973. Seven members of the White Rose had to pay for their bravery with their lives.

## Crossing paths beyond death

**Fig. 9:**
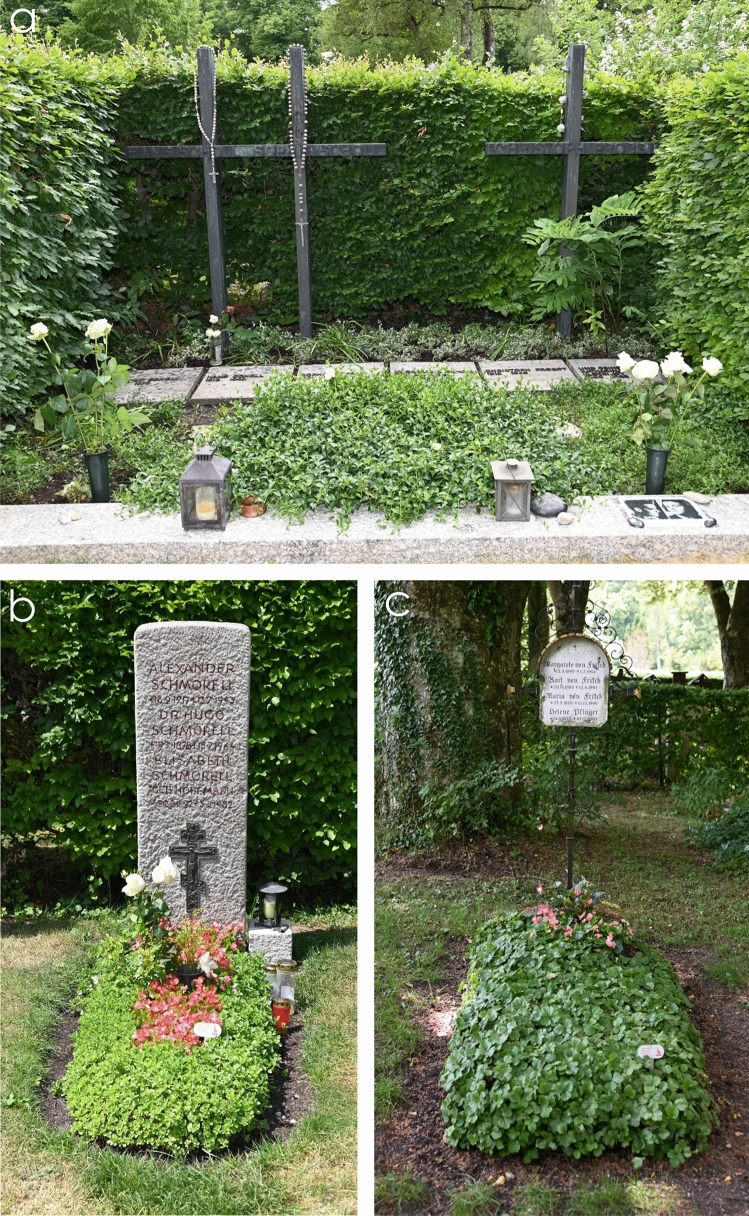
Grave sites of **a** Hans Scholl, Sophie Scholl, and Christoph Probst; **b** Alexander Schmorell; and **c** Karl von Frisch in the Perlacher Forst Cemetery. Photographs by Günther K.H. Zupanc

As an irony of history, the crossing of the paths of Karl von Frisch and members of the White Rose did not stop with their deaths. Several members of the White Rose were laid to rest in the cemetery at *Perlacher Forst* in Munich, after they had been executed in the directly adjacent Stadelheim Prison: Hans Scholl, Sophie Scholl, and Christoph Probst in a joint grave (Fig. 9a), and Alexander Schmorell in a separate grave^84^ (Fig. 9b). Karl von Frisch died on June 12, 1982. He was buried in the same cemetery (Fig. 9c). Remarkably, although the *Perlacher Forst* cemetery extends about 800 m from both north to south and east to west, the graves of von Frisch and Schmorell each are less than 50 m from the Scholl grave.

## Data Availability

Data sharing not applicable to this article as no datasets were generated or analyzed during the current study.

## Abbreviations

BArch: Bundesarchiv Berlin-Lichterfelde
BayHStA: Bayerisches Hauptstaatsarchiv
BSB: Bayerische Staatsbibliothek
UAM: Universitätsarchiv München

## Footnotes

1 Alfred Richard Wilhelm Kühn (1885–1968) was a German zoologist and geneticist who pioneered the integration of the experimental study of development and heredity. His textbooks were for many years among the most widely adopted introductions into zoology and genetics.

2 Konrad Lorenz (1903–1989) was an Austrian zoologist and a co-founder of ethology. He shared the Nobel Prize for Physiology or Medicine in 1973 with Karl von Frisch and Niko Tinbergen.

3 Niko Tinbergen (1907–1988) was a Dutch zoologist and a co-founder of ethology. He shared the Nobel Prize for Physiology or Medicine in 1973 with Karl von Frisch and Konrad Lorenz.

4 Karl von Frisch discovered in the late 1930s and early 1940s that minnows (*Phoxinus phoxinus*) release a substance upon mechanical injury of the skin by a predator. This substance elicits a *Schreckreaktion* (fright reaction) as an anti-predator behavior in other, nearby minnows. He called the substance, which is produced by club cells in the epidermis, *Schreckstoff* (alarm substance). Similar fright reactions have also been found in other Ostariophysi.

5 Karl Wilhelm Theodor Richard von Hertwig (1850–1937) was a German biologist who made important contributions to cytology, embryology, and protozoology.

6 Highest-ranked professor in Germany. As a chair holder in a subject area (e.g., zoology), the *Ordinarius* defined the teaching of this area and was line-manager of lower-ranked professors affiliated with him. The title was abolished as part of a reform of higher education in the 1970s.

7 In Nazi terminology, a quarter-Jew (regardless of religious affiliation or self-identification) was a person with one Jewish grandparent.

8 BayHStA MK 54482.

9 The need for a closer examination of Karl von Frisch and his work during the Nazi era became apparent when the city council of Würzburg, in consultation with the University of Würzburg, renamed the Karl-Ritter-von-Frisch-Weg into Barbara-Thein-Weg in 2023 (https://www.med.uni-wuerzburg.de/aktuelles/meldungen/single/news/eine-strasse-fuer-barbara-thein/; accessed 28 January 2024). This decision was based on conclusions drawn by the advisory board of the city, which found that von Frisch had made statements that they regarded as “unethical,” due to their “eugenic, social Darwinistic, and racist ideas” (https://www.wuerzburg.de/media/www.wuerzburg.de/org/med_515330/574033_abschlussbericht_inkl._anlagen.pdf; accessed 28 January 2024). Karl von Frisch had expressed such views in several editions of his popular scientific book *Du und das Leben* (for further information about this issue see section ‘The political strategy of Karl von Frisch during the Nazi era’ in this article).

10 Richard Martin Willstätter (1888–1991) was a German Jew. His work on the structure of plant pigments, including chlorophyll, won him the Nobel Prize for Chemistry in 1915.

11 Erich Ludendorff (1865–1937) was a German general who is credited for his role in the German victories at Liège and Tannenberg during World War I. However, after the war, he refused to accept any responsibility for Germany’s defeat and, instead, claimed that the Imperial German Army had lost the war not on the battlefield but because it had been betrayed on the home front by communists, socialists, and Jews. This *Dolchstoßlegende* (stab-in-the-back myth) played a central role in the propaganda used by right-wing parties, including the Nazi party, as part of their assault on the Weimar Republic.

12 The *Sturmabteilung* (Assault Division), better known under its abbreviation ‘SA,’ was founded by Adolf Hitler in Munich in 1921 as a paramilitary wing of the Nazi party. Its members were notorious for their violent intimidation of voters, Jews, and political opponents. By use of this instrument, the SA played a key role in Hitler’s rise to power.

13 Sophie Scholl, born Sofie Lina Scholl, used consistently ‘Sophie’ after 1942 (Hockert HG, History und memory: Sophie Scholl in der biographischen Forschung und im Boom der Erinnerung. In: Berg M, Kuchler C (2022) @ichbinsophiescholl. Darstellung und Diskussion von Geschichte in Social Media, pp. 21–39. Wallstein Verlag, Göttingen).

14 The language of these leaflets is very academic, and Schmorell and Scholl frequently made reference to classical literature.

15 Third leaflet; https://www.weisse-rose-stiftung.de/widerstandsgruppe-weisse-rose/flugblaetter/iii-flugblatt-der-weissen-rose/; accessed 9 November 2023.

16 Fifth leaflet; https://www.weisse-rose-stiftung.de/widerstandsgruppe-weisse-rose/flugblaetter/v-flugblatt-der-weissen-rose/; accessed 9 November 2023.

17 Ibid.

18 The Gestapo, an abbreviation of *Geheime Staatspolizei* (Secret State Police), were the political police of the Third Reich.

19 Roland Freisler (1893–1945) was a German judge and politician. He was president of the People’s Court (a special court with jurisdiction over political offenses) from 1942 until 1945. Freisler was killed when the court building was hit by a bomb during a U.S. air raid. He was notorious for humiliating defendants during trials and for frequently issuing the death penalty.

20 The 31 core members and supporters of the White Rose stood trial before the People’s Court in Munch on February 22, 1943 (3 defendants), before the People’s Court in Munich on April 19, 1943 (14 defendants), before the *Sondergericht beim Landesgericht München* (‘Special Court at the State Court Munich’) on July 13, 1943 (4 defendants), before the *Landesgericht Saarbrücken* (‘State Court Saarbrücken’) on January 28, 1944 (1 defendant), and before the People’s Court in Donauwörth on October 13, 1944 (9 defendants). Details provided by Hans Günter Hockerts via email to GKHZ on February 9, 2024.

21 In Nazi terminology, a half-Jew was (regardless of religious affiliation or self-identification) a person with two Jewish grandparents.

22 Despite his status as a ‘half-Jew,’ Hans Conrad Leipelt was employed by Heinrich Otto Wieland (1877–1957), a professor of chemistry at the University of Munich who even appeared as a witness for the defense at Leipelt’s trial before the People’s Court in Donauwörth. Wieland was one of the leading chemists of his time. During World War I, he was involved in chemical warfare research at the Kaiser Wilhelm Institute for Physical Chemistry and Electrophysiology in Dahlem. In 1925, he succeeded Richard Willstätter as *Ordinarius* at the University of Munich. In 1927, he was awarded the Nobel Prize in Chemistry for his studies on the constitution of bile acids and related substances. Wieland’s research led to many breakthroughs in organic chemistry and biochemistry. During the Third Reich, he accommodated, as ‘personal guests,’ and funded through third-party projects, approximately 25 students (most of them half-Jews) who had been expelled from the University of Munich and other academic institutions in Germany. This arrangement, and the esteem he held with the Nazi regime despite his frequent ambiguous remarks and non-conformist behavior, provided these students, to a certain degree, with shelter from the Nazis. For historical reviews see Vaupel E (2007) Interconnections and independence: Heinrich Wieland (1877–1957) and his era. Angew. Chem Int. Ed. 46:9154-9179. https://doi.org/10.1002/anie.200702255; Witkop B (2008) Principiis obsta: Erinnerungen an Heinrich Wieland. In: Wieland S, Hertkorn A-B, Dunkel F (eds) Heinrich Wieland. Wiley–VCH, Weinheim.

23 In this article, ‘course enrollment’ refers to formal registration for a class and payment of *Hörergeld* (tuition fee), as documented in the *Belegblatt* (course registration card) of the respective student at the University of Munich.

24 UAM Stud-BB-1253 (SoSe 1942).

25 Friedrich Carl von Faber (1880-1954), Director of the Botanical Garden and *Ordinarius* of Botany at the University of Munich.

26 Gottfried Wilhelm von Leibniz (1646–1716), German mathematician, philosopher, scientist, and diplomat.

27 In her book, Beuys does not indicate the source of this information. We contacted her, but she was unable to provide us with the source because, according to her response, she destroyed the documents on which her book is based. Our own attempt to find Sophie Scholl’s *Belegblatt* (course registration card) of the winter semester 1942/1943 in the Archive of the University of Munich or elsewhere has remained unsuccessful. Thus, we are neither able to confirm that Sophie Scholl took a course with Karl von Frisch during this semester nor can we, if she did indeed take a course, specify which course it was.

28 UAM Stud-BB-1137 (Hans Scholl) and UAM Stud-BB-1142 (Christoph Probst). The information on the course registration forms is ambiguous. Hans Scholl entered on his form for course instructor ‘v. Frisch,’ for course name ‘Zoologie I,’ for credit hours ‘3,’ and for tuition fee ‘9’ (Reichsmark). Christoph Probst entered on his form for course instructor ‘v. Frisch,’ for course name ‘Zoologie,’, for credit hours ‘2,’ and for tuition fee ‘9’ (Reichsmark). According to the *Vorlesungsverzeichnis* (course listing) of the summer semester 1939 (https://epub.ub.uni-muenchen.de/827/1/vvz_lmu_1939_sose.pdf; accessed 16 February 2024), von Frisch taught during this term ‘Zoologie II’ (Zoology Lecture Course II) and the ‘Zoologischer Kurs für Mediziner’ (Zoological Lab Course for Medical Students). The latter was taught jointly with Werner Jacobs.

29 Werner Jacobs (1901–1972) studied sciences at the University of Rostock and earned a Ph.D. under the mentorship of Karl von Frisch in 1924. In 1925, he joined von Frisch as assistant at the University of Munich, where he received his *Habilitation* in 1930. In 1937, he was appointed to an adjunct professorship, and in 1939 to a tenured *Konservator* position at the Zoological Institute of the University of Munich. The focus of his research was mainly on the ethology and physiology of insects. Jacobs was a member of eight Nazi organizations, including the Nazi party (1937–1945). He served as an *Oberscharführer* (senior squad leader) in the SA during the entire Nazi era, from 1933 to 1945. In 1946, he was suspended from his position because of his membership in the NSDAP, but in 1948 he was reinstated (UAM FakBio-IX-28).

30 After the beginning of World War II on September 1, 1939, an increasing number of male students were drafted into the military. To offset the expected shortage of graduates from German universities, effective fall 1939 the duration of college education was reduced by replacing the semester system by a trimester system. After this effort failed to achieve the desired goal, the semester system was reactivated in the winter semester 1941/1942.

31 In the course listing of the winter semester 1939/1940, Werner Jacobs is listed as sole instructor of this course. However, he was called up for military service, effective August 26, 1939 (UAM E-II-1855). It is, therefore, likely that Karl von Frisch served as a substitute instructor for the Comparative Anatomy of Vertebrates lab course during this term. This assumption is consistent with the fact that Scholl, on his registration form, listed von Frisch as instructor of this course.

32 UAM Stud-BB-1164 (Hans Scholl), UAM Stud-BB-1167 (Christoph Probst), and UAM Stu-BB-1167 (Alexander Schmorell).

33 This letter was written one day after Sophie Scholl’s execution. At that time, Fritz Hartnagel was not yet aware of the arrest, trial, and execution of Hans and Sophie Scholl, as well as Christoph Probst.

34 Lviv, a city in western Ukraine, was known as Lemberg during the Austro-Hungarian Empire and the German occupation between 1941 and 1944.

35 Elisabeth Hartnagel (née Scholl) was the sister of Sophie Scholl. She married Sophie’s former fiancée Fritz Hartnagel in October 1945.

36 Inge Scholl (1917–1998) was the eldest sibling of Hans and Sophie Scholl. In 1952, she married the graphic designer and typographer Otl Aicher, and since then became known as ‘Inge Aicher-Scholl.’ In 1952, she published the book *Die Weiße Rose* (The White Rose), in which she portrayed Sophie Scholl, beside Hans Scholl, as a central figure of the White Rose anti-Nazi resistance group. However, while Inge Scholl’s book made Sophie Scholl an icon of the anti-Nazi resistance, there is consensus among historians that, overall, Sophie played a rather peripheral role in the White Rose group (Hikel C (2012): Sophies Schwester. Inge Scholl und die Weiße Rose. R. Oldenbourg Verlag, München).

37 The *Volkhochschulen* (adult education centers) provide continued education to adult learners in Germany, without offering the opportunity to earn credits for college.

38 While Sophie Scholl indeed attended Karl von Frisch’s General Zoology during the summer semester of 1942, there is no evidence that Hans Scholl, who enrolled at the University of Munich three years earlier, took any classes with him during that year. According to the records we retrieved from the University Archive, he was registered for two of his classes during his freshman year, in 1939.

39 BSB Ana-540, B.VI.8 Ulmer Volkshochschule.

40 Jens (2021, p. 30).

41 Hans Krieg (1888–1970) studied medicine at the University of Tübingen and zoology at the University of Munich, and earned both a Ph.D. and an M.D. In 1921, he received his *Habilitation* from the University of Tübingen. In 1927, he was appointed director of the zoological collections of the State of Bavaria and adjunct professor of zoology at the University of Munich. Between 1945 and 1957, he was director general of the scientific collections of the State of Bavaria. In the 1920s and 1930s, he went on several scientific expeditions to South America. Krieg was a member of the Nazi party from 1937 to 1945 (UAM FakBio-IX-43).

42 Thirty-five recipients of leaflets 1, 2, 3, and/or 4 of the White Rose handed in their copies to the Gestapo. Individual recipients reported that they had received between one and four leaflets. In their list (BArch R 3017/34635), the Gestapo documented the name, profession, residential address of each recipient, and, for each leaflet, the location of the post office that had postmarked the stamps on the envelope in which the leaflet was mailed, as well as the postmark date and time. A copy of the Gestapo list was kindly provided by Hans Günter Hockerts.

43 Kronawitter, H. (2013) 25 Jahre Weiße Rose Stiftung e.V.—ein Beitrag zur Erinnerungskultur. Einsichten und Perspektiven 1, 6–17.

44 In 1946, the plaque was mounted next to the entrance to the main assembly hall. In 1957, it was moved to the wall on the northern upper gallery, where Sophie Scholl is believed to have dropped copies of the sixth pamphlet into the inner courtyard (cf. https://www.tracesofevil.com/search/label/Wei%C3%9Fe%20Rose; accessed 16 July 2023).

45 The perhaps most prominent artistic piece is the pavement memorial to the White Rose in front of the main entrance of the university building (Fig. 7). It was created by Robert Schmidt-Matt in 1990, i.e., it was unknown to Karl von Frisch, who passed away in 1982.

46 The frequent focus in the remembrance of the White Rose on the Scholl siblings, and on Sophie Scholl in particular, has clearly resulted in a distortion of the historical contributions (and sacrifices) of the other members. For example, as of November 2010, 188 schools in Germany were named after Sophie Scholl, Hans Scholl, or both (Matthias Kalle: Gedenkschule. ZEITmagazin 4. November 2010). Conversely, according to a Google search on July 16, 2023, only one school, the *Fach- und Berufsoberschule* in Donauwörth, is named after Hans Conrad Leipelt.

47 Personenstand der Universität München, abgeschlossen am 15. Oktober 1942 (https://epub.ub.uni-muenchen.de/9733/1/pvz_lmu_1942_wise_sose.pdf; accessed 16 July 2023).

48 The study of botany and zoology was not divided into undergraduate and graduate study programs. Students either completed a Ph.D. thesis (typically in the 4th year of study) or took the *Prüfung für das höhere Lehramt* (state exam for teaching at high schools) as the terminal exam. Between 1930 and 1942, the percentage of zoology and botany students among the science students who were awarded a Ph.D. ranged from 15.6 to 30.9%. Although detailed figures are not available, indirect evidence suggests similar percentages for students who took the state exam for teaching at high schools.

49 Julian von Frisch (born 1965) is the son of Otto von Frisch. The latter was the only boy among the four children of Karl von Frisch. Julian von Frisch knew his grandfather mostly from the time he spent with him during the family’s summer vacation in Brunnwinkl.

50 The interview of GKHZ and SW with Julian von Frisch took place via a Zoom video conference on June 8, 2023.

51 Besides members of the White Rose, this ignorance extends to other students who were enrolled in his classes and later became figures of historical significance. One of them is Josef Mengele (1911–1979), a German physician, anthropologist, and SS officer. He is most infamous for his pseudoscientific experiments on inmates that often resulted in the death of the subject and for his role in selecting victims for killing in the gas chamber in Birkenau, the supplementary extermination camp at Auschwitz. After World War II, Mengele was one of the most-wanted Nazi criminals. During the winter semester of 1934/1935, he took Zoology and the Comparative Physiological Lab with Karl von Frisch (UAM Stud-BB-1048).

52 Karl von Frisch’s willingness to come to terms with the Nazi regime included his frequent ending of letters to the rector of the University of Munich and to authorities of the Reich Government with ‘Heil Hitler.’ On the other hand, according to the *Hochschullehrerkartei* (card index of university professors) of the Reich Ministry of Science, Education, and National Culture in Berlin, he was never a member of any Nazi organization, including the Nazi party (BArch R4901/13263). For comparison, of the zoologists who did not emigrate during the Third Reich, 55% had joined the Nazi party (Deichmann U (1996) Biologists under Hitler. Harvard University Press, Cambridge, Massachusetts). Among Karl von Frisch’s family, his brother Hans von Frisch, an Austrian professor of constitutional law, became a member of the Nazi party on March 1, 1933—just five weeks after Adolf Hitler had been appointed Chancellor. However, he was expelled in 1939, after it was discovered that his maternal grandmother was a Jew.

53 Eugenics was a popular concept at the time when Karl von Frisch published his book Man and the Living World—not just in Germany but also in other countries. The term eugenics was coined by the British polymath Francis Galton in 1883. He advocated for a scientific strategy to “improve stock” and, especially in the case of humans, “to give the more suitable races or strains of blood a better chance of prevailing speedily over the less suitable than they otherwise would have had.” By the beginning of the twentieth century, the idea of improving ‘superior’ human races through selective breeding had become a popular idea in many countries. It gained support not only from scientists, such as the American geneticist and Nobel laureate Hermann Joseph Muller, but also from leading politicians, including the U.S. President Theodore Roosevelt. A central figure in promoting this idea in the United States was Harry H. Laughlin, who was superintendent of the Eugenics Record Office at Cold Spring Harbor, funded to a large part by the Rockefeller Foundation and the Carnegie Institution. He drafted a model law to control reproduction through mandatory sterilization of what he considered genetically and socially inferior institutionalized individuals. It was adopted subsequently by more than half of the states and led to 70,000 forced sterilizations during the twentieth century. Core elements of Laughlin’s law were implemented by Nazi Germany in the *Gesetz zur Verhütung erbkranken Nachwuchses* (Law for the Prevention of Hereditary Diseased Offspring), which took effect in January 1934. Adolf Hitler praised the American eugenics programs, and several of the leading American eugenicists expressed their admiration for the eugenical laws of the Third Reich. In the Weimar Republic, implementation of eugenic measures was discussed by various parties, including the Social Democratic Party, but even legislation ‘just’ for voluntary sterilization did not pass any parliament. The initial support for the Nazi eugenics program by significant parts of the United States rapidly declined when the magnitude of forced sterilizations in Germany became known. As a result, the Carnegie Institution closed the Eugenics Record Office in 1939. Nevertheless, forced surgical sterilization of children with disabilities is still allowed in about one third of the U.S. states.

54 Aryanization was the expropriation of Jewish property, often below market value, by the Third Reich. It started within a few months after Adolf Hitler took office as chancellor of Germany on January 30, 1933.

55 We had two Deutsche Verlag editions available for evaluation. One edition, indicating only the copyright date of 1936, and the number of copies printed (56,000–61,000), had the label *Dr.-Goebbels-Spende für die deutsche Wehrmacht* printed on the front cover and the book spine. The other edition, again showing the copyright date of 1936 but also a print date (1940) and the number of copies printed (21,000–30,000), did not have the *Dr.-Goebbels-Spende* label. Funding through the *Dr.-Goebbels-Spende für die Deutsche Wehrmacht* of the Deutsche Verlag 1936 edition is also mentioned by Tania Munz in her biography, The Dancing Bees: Karl von Frisch and the Discovery of the Honeybee Language (University of Chicago Press, Chicago 2016).

56 Joseph Goebbels (1897–1945), as Reich Minister of Propaganda from 1933 to 1945, was one of the most influential Nazi officials of the Third Reich and perhaps the closest confident of Adolf Hitler.

57 Without explicitly mentioning the 1934 Law for the Prevention of Hereditary Diseased Offspring in this part of the book, it is obvious that von Frisch makes reference to this law. It mandated forcible sterilization for people suffering from eight hereditary disorders, including schizophrenia, Huntington disease, genetically caused blindness and deafness, and severe physical disabilities. In addition, sterilization was also legitimized of individuals with severe alcoholism. Supplementary provisions over the following years added castration and forcible abortions to the list of legal procedures. Under this law, approximately 400,000 people were sterilized until 1945. About 5,500 women and 600 men died from complications during and after the procedures. The ideology of promotion of selective breeding of *Höherwertige* (superior people) and the exclusion of *Minderwertige* (inferior people) from reproduction, which provided the foundation for the eugenic measures implemented in the Law for the Prevention of Hereditary Diseased Offspring, was also used as a pseudo-scientific justification for the clandestine *Aktion T4*, a major euthanasia program resulting in the murdering of 300,000 people during the Third Reich.

58 The *Rassenpolitische Amt* (Office of Racial Policy) was a department of the Nazi party. Its major role was to promote eugenics, for example by emphasizing the importance of race and health for marriage.

59 *Das Reich* was a weekly newspaper founded by Joseph Goebbels in 1940. Its circulation grew to over 1.4 million by 1944, making it one of the most-read newspapers during the Nazi era. Goebbels himself contributed a weekly editorial.

60 BayHStA MK 43414.

61 BSB Ana-540, C.III.23.

62 Leo von Zumbusch. https://de.wikipedia.org/wiki/Leo_von_Zumbusch. Accessed 6 August 2023.

63 Nora Exner. https://de.wikipedia.org/wiki/Nora_Exner. Accessed 6 August 2023.

64 Adolf Exner. https://de.wikipedia.org/wiki/Adolf_Exner. Accessed 6 August 2023.

65 This comment in Karl von Frisch’s letter is notable because Konrad Lorenz denied his membership in the Nazi party throughout his life (Taschwer, K.: Die verlorene Ehre des Konrad Lorenz. Der Standard, December 18, 2015). However, the discovery of his membership card in the archives of the Berlin Document Center (Kalikow 1983) not only has proven that he was a member but also has shown that he applied for membership as early as May 1, 1938—just seven weeks after the Anschluss. Further insights into his sympathies for National Socialism can be gained through the letter of motivation that Lorenz submitted as part of his party membership application. “As someone who thinks German and is a scientist, it is natural that I always have been a national socialist… I have devoted my entire scientific career, which focuses on topics related to evolution, race, and social psychology, to the ideology of national socialism” (Föger and Taschwer 2001, p. 79).

66 BSB Ana-540, C.III.74.

67 In sharp contrast to Konrad Lorenz, Niko Tinbergen vehemently opposed the Nazi regime. In 1942, when the Nazi occupation forces in The Netherlands demanded the expulsion of Jews from the University of Leiden, Tinbergen and other faculty members refused to follow the order. As one of the ringleaders of this action, he was arrested and, together with several hundred Dutch hostages, detained in the Roman Catholic minor seminary Beekvliet in the North Brabant municipality of Sint-Michielsgestel. There, he lived for the following two years under the constant threat of execution in retaliation for acts of sabotage by the Dutch Resistance Movement.

68 BSB Ana-540, C.III.74.

69 Rudolf Werner Georg Hippius (1905–1945) was a Baltic-German psychologist.

70 Lorenz (1940, p. 66)

71 Lorenz (1940, pp. 66 and 68)

72 Lorenz (1940, pp. 71–72)

73 The *Nürnberger Gesetze* (Nuremberg Laws) were two laws enacted in 1935 during the annual Nuremberg Rally of the Nazi Party. The *Reichsbürgergesetz* (Reich Citizen Act) limited Reich citizenship to subjects of the state that were of German or related blood. The *Blutschutzgesetz* (Blood Protection Act) forbade marriages and extramarital relations between Jews and citizens of German and related blood.

74 This letter is not preserved in Karl von Frisch’s estate. However, based on von Frisch’s reference to the date of Simon Wiesenthal’s letter, and to its content, it is evident that the letter to von Frisch was one of the several hundred identical copies (all dated November 2, 1964) mailed to the ‘intellectual elite’ of Germany and Austria. The template of this letter can be found on pages 7–9 in Wiesenthal S (ed.) Verjährung? 200 Persönlichkeiten des öffentlichen Lebens sagen NEIN—Eine Dokumentation von Simon Wiesenthal. Europäische Verlagsanstalt, Frankfurt am Main (1965).

75 Simon Wiesenthal (1908–2005), an architectural engineer by training, was a prisoner in five Nazi-concentration camps during World War II. After the War, he dedicated his life to the search for Nazi criminals, in part as head of the Documentation Center of the Association of Jewish Victims of the Nazi Regime in Vienna.

76 The prolongation of the limitations period to Nazi crimes was based on the argument that German courts had been incapacitated during the first few years after Germany’s unconditional surrender on May 8, 1945. The constitutionality of this retroactive measure was, however, doubtful and led to the resignation of the Secretary of Justice, Ewald Bucher.

77 BArch R 4901/24579.

78 The *Dozentenschaft* (Association of University Lecturers) and the *Nationalsozialistischer Deutscher Dozentenbund* (National Socialist German University Lecturers League) played important roles during the Nazi regime in ensuring through their evaluation of candidates that faculty were appointed not just based on scientific merit but also on their conformity with Nazi ideology. Wilhelm Führer (1904–1974) and Ernst Bergdolt (1902–1948) were leading figures of these two organizations in Munich.

79 Otto Egon Löwenstein (1906–1999), born in Munich, was half Jew and half Roman Catholic. He started working with von Frisch as an undergraduate student, under whose mentorship he also carried out his Ph.D. thesis research on the equilibrium organs in minnows. As a half Jew, he lost his research grant after the Nazis had gained power, but Karl von Frisch paid him a salary from a ‘special source’ (which, as Löwenstein suspected later, might have come from von Frisch’s own pocket). In October 1933, Löwenstein emigrated to Great Britain, where his research contributed much to our understanding of the physiology of the organs of equilibrium.

80 Curt Stern (1902–1981) was born into a Jewish family in Hamburg. He received his Ph.D. in 1923 for his study of the reproduction of the protozoan *Heliozoa* in the lab of Max Hartmann at the Kaiser Wilhelm Institute for Biology in Berlin. Between 1924 and 1926, he studied genetics in the lab of Thomas Hunt Morgan at Columbia University in New York. During the subsequent years at the University of Berlin, he established a highly successful research program on *Drosophila* genetics. In 1932, he went to the United States for a research visit to the California Institute of Technology. After Hitler had come to power, he decided not to return to Germany. In the United States, Stern established a highly successful scientific career, first at Case Western Reserve University, then at the University of California, Berkeley.

81 Dorothea ‘Dora’ Ilse (1898–1979), born in Honnef am Rhein, was of Jewish origin (details unknown). She received her Ph.D. in 1928 for a study on color vision in butterflies, conducted in the lab of Alfred Kühn at the University of Göttingen. From 1931 to 1932, she was a scientific assistant in the lab of Richard Goldschmidt at the Kaiser Wilhelm Institute for Biology in Berlin. From 1932 to 1935, she worked at the Zoological Institute of the University of Munich as a *private Hilfskraft*, a term indicating that, while holding a low-level position, she did not receive any salary. In 1935, she emigrated to Great Britain. In 1950, she returned to Germany. Ilse translated Karl von Frisch’s book *Aus dem Leben der Bienen* into English, which appeared in its first edition under the title ‘The Dancing Bees’ in 1955.

82 In a *Sonderaktion* (special operation), the Gestapo arrested 183 faculty members, including 22 life scientists, from the universities in Krakow (German: *Krakau*) in occupied Poland on November 6, 1939. Their arrest was part of Germany’s plan to eliminate intellectuals and other members of Poland’s ‘leadership class.’ Among those arrested was the biologist Roman J. Wojtusiak (1906–1987) who, in 1932, had spent a year as a visiting scholar in the laboratories of Alfred Kühn in Göttingen and Karl von Frisch in Munich. He was first deported to the concentration camp (German *Konzentrationslager* or *Konzentrationszuchthaus*, KZ) in Sachsenhausen, and then to the KZ in Dachau. In early 1940, Karl von Frisch received a letter from Halina Wojtusiakowa, the wife of Roman Wojtusiak, in which she asked for his help. Over the following months, von Frisch successfully invoked the support of Walter Greite (1907–1984), an SS officer and Nazi bureaucrat who had conducted his Ph.D. thesis under Alfred Kühn. In September 1940, Wojtusiak was released and returned to Krakow. For a detailed account of the *Sonderaktion Krakau 1939* and the role of Karl von Frisch in the release of Roman J. Wojtusiak, see Seyfarth E-A, Pierzchala H (1992) Sonderaktion Krakau 1939: Die Verfolgung von polnischen Biowissenschaftlern und Hilfe durch Karl von Frisch. Biologie in unserer Zeit 22:218–225. https://doi.org/10.1002/biuz.19920220424.

83 Oberwittler D (2015) Kriminalität. In Rahlf T (ed) Deutschland in Daten. Zeitreihen zur Historischen Statistik, pp 142–154. Bundeszentrale für politische Bildung, Bonn. http://www.bpb.de/shop/buecher/zeitbilder/211002/deutschland-in-daten.

84 Hans Conrad Leipelt’s remains are buried in an anonymous grave commemorating victims of Nazi crimes in the *Perlacher Forst* cemetery.
